# GFAP Isoforms in Adult Mouse Brain with a Focus on Neurogenic Astrocytes and Reactive Astrogliosis in Mouse Models of Alzheimer Disease

**DOI:** 10.1371/journal.pone.0042823

**Published:** 2012-08-13

**Authors:** Willem Kamphuis, Carlyn Mamber, Martina Moeton, Lieneke Kooijman, Jacqueline A. Sluijs, Anne H. P. Jansen, Monique Verveer, Lody R. de Groot, Vanessa D. Smith, Sindhoo Rangarajan, José J. Rodríguez, Marie Orre, Elly M. Hol

**Affiliations:** 1 Netherlands Institute for Neuroscience - an Institute of the Royal Netherlands Academy of Arts and Sciences (KNAW), Department of Astrocyte Biology & Neurodegeneration, Amsterdam, The Netherlands; 2 Swammerdam Institute for Life Sciences, Center for Neuroscience, University of Amsterdam, Amsterdam, The Netherlands; 3 Mayo Clinic College of Medicine, Rochester, Minnesota, United States of America; 4 IKERBASQUE, Basque Foundation for Science, Bilbao, Spain/Department of Neurosciences, University of the Basque Country UPV/EHU, Leioa, Spain; Boston University School of Medicine, United States of America

## Abstract

Glial fibrillary acidic protein (GFAP) is the main astrocytic intermediate filament (IF). GFAP splice isoforms show differential expression patterns in the human brain. GFAPδ is preferentially expressed by neurogenic astrocytes in the subventricular zone (SVZ), whereas GFAP^+1^ is found in a subset of astrocytes throughout the brain. In addition, the expression of these isoforms in human brain material of epilepsy, Alzheimer and glioma patients has been reported. Here, for the first time, we present a comprehensive study of GFAP isoform expression in both wild-type and Alzheimer Disease (AD) mouse models. In cortex, cerebellum, and striatum of wild-type mice, transcripts for Gfap-α, Gfap-β, Gfap-γ, Gfap-δ, Gfap-κ, and a newly identified isoform Gfap-ζ, were detected. Their relative expression levels were similar in all regions studied. GFAPα showed a widespread expression whilst GFAPδ distribution was prominent in the SVZ, rostral migratory stream (RMS), neurogenic astrocytes of the subgranular zone (SGZ), and subpial astrocytes. In contrast to the human SVZ, we could not establish an unambiguous GFAPδ localization in proliferating cells of the mouse SVZ. In APPswePS1dE9 and 3xTgAD mice, plaque-associated reactive astrocytes had increased transcript levels of all detectable GFAP isoforms and low levels of a new GFAP isoform, Gfap-ΔEx7. Reactive astrocytes in AD mice showed enhanced GFAPα and GFAPδ immunolabeling, less frequently increased vimentin and nestin, but no GFAPκ or GFAP^+1^ staining. In conclusion, GFAPδ protein is present in SVZ, RMS, and neurogenic astrocytes of the SGZ, but also outside neurogenic niches. Furthermore, differential GFAP isoform expression is not linked with aging or reactive gliosis. This evidence points to the conclusion that differential regulation of GFAP isoforms is not involved in the reorganization of the IF network in reactive gliosis or in neurogenesis in the mouse brain.

## Introduction

Astrocytes have a variety of functions in the brain providing general structural, metabolic, and trophic support to neurons [1]. In addition to these functions, astrocytes are also actively involved in modulating normal synaptic transmission [2], [3]. Different subsets of astrocytes have been described, probably with different and specific functions [4]. A special subset is formed by astrocytes that have been identified as adult neural stem cells in the two main neurogenic niches in the adult brain, the hippocampal subgranular zone (SGZ), and the subventricular zone (SVZ) [5].

In response to damage inflicted to the central nervous system, astrocytes change from their normal quiescence into a so-called reactive state. This process of reactive gliosis is characterized by morphological changes (hypertrophy), functional alterations, and by a profound increase in the expression of the astrocyte-specific intermediate filament (IF) glial fibrillary acidic protein (GFAP) [6]–[8]. IFs are now known to be dynamic structures involved in a wide range of cellular processes during homeostasis and stress [9]. Although an increased GFAP expression is widely used as a marker for astrogliosis, the precise functional role of GFAP in astrocytes is not known and the implications of an increased GFAP expression for astrocyte-mediated functions in gliosis have remained elusive [1], [8]. GFAP overexpression and mutations in both the tail and rod domain of the protein influence the motility of glioma cells *in vitro* [10], [11]. Elevated levels of mutated GFAP have been related to an impaired proteasomal and/or autophagy activity [12]–[14]. Surprisingly, GFAP^−/−^ mice show little apparent defects and display reactive gliosis, possibly because of the remaining presence of vimentin [15]. Nevertheless, GFAP^−/−^ mice have been demonstrated to be more sensitive to spinal cord injury [16], to cerebral ischemia [17], [18], and to neurotoxicity [19], indicating a protective role of GFAP. GFAP^−/−^ astrocytes also fail to form a barrier-like structure around amyloid β (Aβ) deposits, suggesting a role for GFAP in the structural alterations of reactive astrocytes surrounding plaques in Alzheimer Disease (AD) [20].

To date, nine splice variants of GFAP are described in different species (human, mouse, rat). The canonical isoform, GFAPα, has nine exons. GFAPβ has an alternative upstream transcriptional start site in the 5′UTR [21], [22]. GFAPγ lacks exon 1 and includes the last 126 bp of intron 1–2 [23]. Three splice variants GFAPΔEx6, GFAPΔ164, and GFAPΔ135 skip sequences in exon 6/7 [24]. In a paper by Zelenika and colleagues, a transcript including the last 284 bp of intron 8–9 was reported, here termed GFAPζ (zeta) [23]. GFAPδ and GFAPκ comprise intron 7–8 sequences [25]–[27]. These splice variants are differentially expressed in the human brain. GFAPΔEx6, GFAPΔ164, and GFAPΔ135 were identified in AD tissue, in focal lesions in chronic epilepsy, and in a specific human astrocyte subtype [24], [28], [29]. The GFAPδ isoform has received most attention, since it is differentially expressed in proliferating cells within the subventricular zone (SVZ) of the human brain, in astrocytes bordering the rostral migratory stream (RMS), and in the olfactory bulb [25], [30], [31]. GFAPδ is also expressed in some neuropathological conditions such as human spinal cord astrocytoma [32], [33], [34], Vanishing White Matter [35], and after ischemic stroke [36]. Why certain GFAP isoforms are associated with subtypes of astrocytes, high-grade astrocytomas or other pathophysiological conditions is not known, but it has been speculated that the variable C-terminal regions affect the assembly of GFAP filaments. A differential assembly of GFAP filaments lead to changes in the binding of interacting proteins thereby altering cellular functions (such as neurogenesis) or cellular morphology (such as gliosis) [27], [37].

In previous studies, we reported the differential expression of GFAPδ and GFAP^+1^ in distinct types of astrocytes in the human brain [25], [29], [31], but a detailed description of GFAP isoform expression in mouse brain has not been published so far. In the current study we describe the generation of mouse GFAP isoform specific qPCR assays and the making of specific antibodies against mouse GFAPδ, GFAPκ, and GFAP (GFAP^+1^) isoforms Gfap-ΔEx6 and Gfap-Δ164. The splicing out of 221 bp or 164 bp results in a shifted reading frame and GFAP proteins which lack part of coil 2B and the entire tail region and an unique C-terminal epitope [24]. The *first* goal of our study was to investigate the GFAP isoform expression in mouse brain with emphasis on GFAPδ to address whether this isoform is, comparable to human GFAPδ, associated with neurogenic astrocytes in the SVZ. The *second* goal was to assess the changes in GFAP isoform expression in AD-related reactive gliosis to test the hypothesis that a differential GFAP isoform expression may be involved in the morphological changes during gliosis, as previously described by our group for human AD [24], [25]. To this end, we determined the changes in transcript levels and protein distribution of GFAP isoforms and other IFs (vimentin, nestin, synemin) at different stages of plaque load in the APPswePS1dE9 and 3xTgAD mouse models. These models are known to have extensive plaque-related gliosis [38]–[40].

## Results

### Development of Isoform Specific qPCR Assays

**Figure 1:**
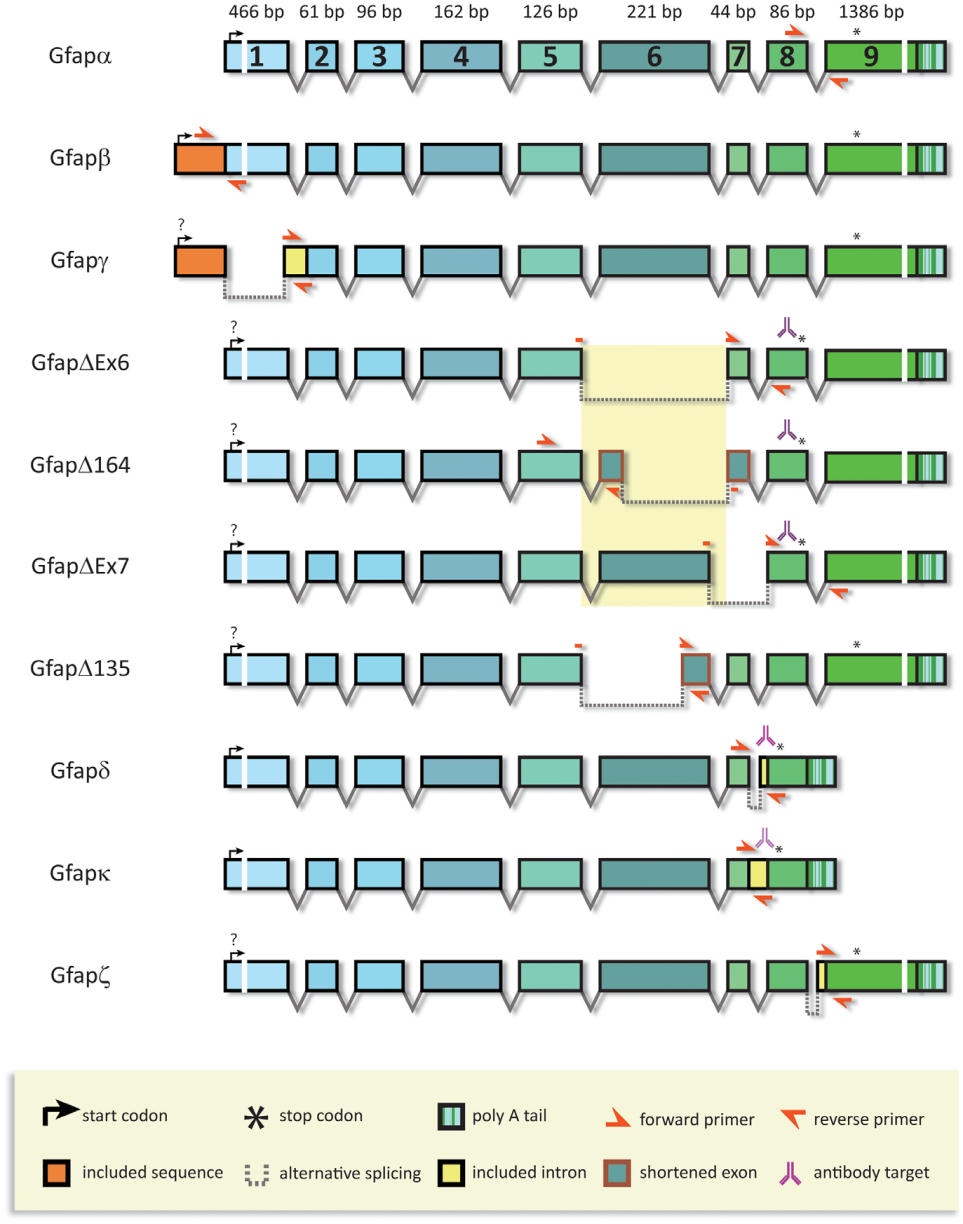
Schematic representation of the different mouse GFAP isoforms studied. The scheme illustrates the differential splicing routes resulting in 10 different Gfap transcript isoforms. The 9 exons containing the canonical Gfap-α isoform is shown on top. Size of the depicted exons is to scale except for exon 1 and 9, indicated by breaks. The target position of primers used for qPCR assays are indicated (see Table S1 for their sequences). The position of the epitope for the isoform-specific antibodies generated by us is indicated. Note that only the full-length sequences of mouse Gfap-α, Gfap-δ [25], and Gfap-κ [26] were identified by us. Transcripts encoding for GfapΔ135, and the GFAP+1 variants (GfapΔ164 and GfapΔEx6), as found in human brain [24], were not detected by qPCR. We found evidence for the existence of GfapΔEx7, a potential GFAP+1 variant, but no effort was made to clone the full-length sequence. Gfap-δ and Gfap-κ each encode for a unique C-terminal amino acid sequence of 41 aa and 46 aa, respectively, different from the Gfap-α encoded C-terminus. Gfap-β was decribed for rat brain [21], Gfap-γ and Gfap-ζ were isolated from mouse brain [23].

Primer design for Gfap specific isoform qPCR assays was based on targeting unique sequences in the different Gfap isoforms. Figure 1 shows an overview of the ten different GFAP isoforms studied illustrating the differences at transcript level, the position of the qPCR primers, and the epitopes for the antibodies used. The design for Gfap-β, Gfap-γ, and Gfap-κ was straightforward and directed against unique sequences. For Gfap-δ, the primers spanned the 390 bp Gfap-κ specific insert and the rapid qPCR cycling conditions never lead to co-amplification of Gfap-κ. Gfap-α has no unique sequences and we designed primers positioned in exon 8 and 9 detecting theoretically all isoforms except Gfap-δ, Gfap-κ and Gfap-ζ, but because the other isoforms are low-abundant we use this assay to determine the Gfap-α mRNA levels. For Gfap-Δex6, Gfap-Δ135, Gfap-Δ164, and Gfap-ΔEx7, one of the primers was positioned over the unique spliced sequence. For these primers, cross-reactivity may occur with Gfap-α as target resulting in the same amplicon. Therefore, assay specificity and sensitivity was tested on serial dilutions of plasmid templates of the different Gfap isoforms (5.10^2^–14.10^7^ copies per PCR reaction). No cross-reaction was found for the specific primers for Gfap-β, Gfap-γ, Gfap-δ, Gfap-κ, Gfap-Δ164, and Gfap-ΔEx7. For Gfap-Δ135 and Gfap-ΔEx6, primer pairs without detectable Gfap-amplification had a much lower sensitivity. For Gfap-ζ, specific primers were positioned in intron 8/9 and exon 9 [23]. On mouse cDNA this resulted in clear amplification, whereas no amplification was found when omitting the reverse transcriptase step or in cDNA from GFAP-knockout mice [15] (data not shown). Using qPCR, we determined the Gfap-isoform expression profiles in different brain areas and in the cortex of aging WT mice, APPswePs1dE9 and 3xTgAD mice.

#### (i) Gfap isoform transcript levels in mouse cortex, cerebellum, hippocampus, and SVZ

**Figure 2:**
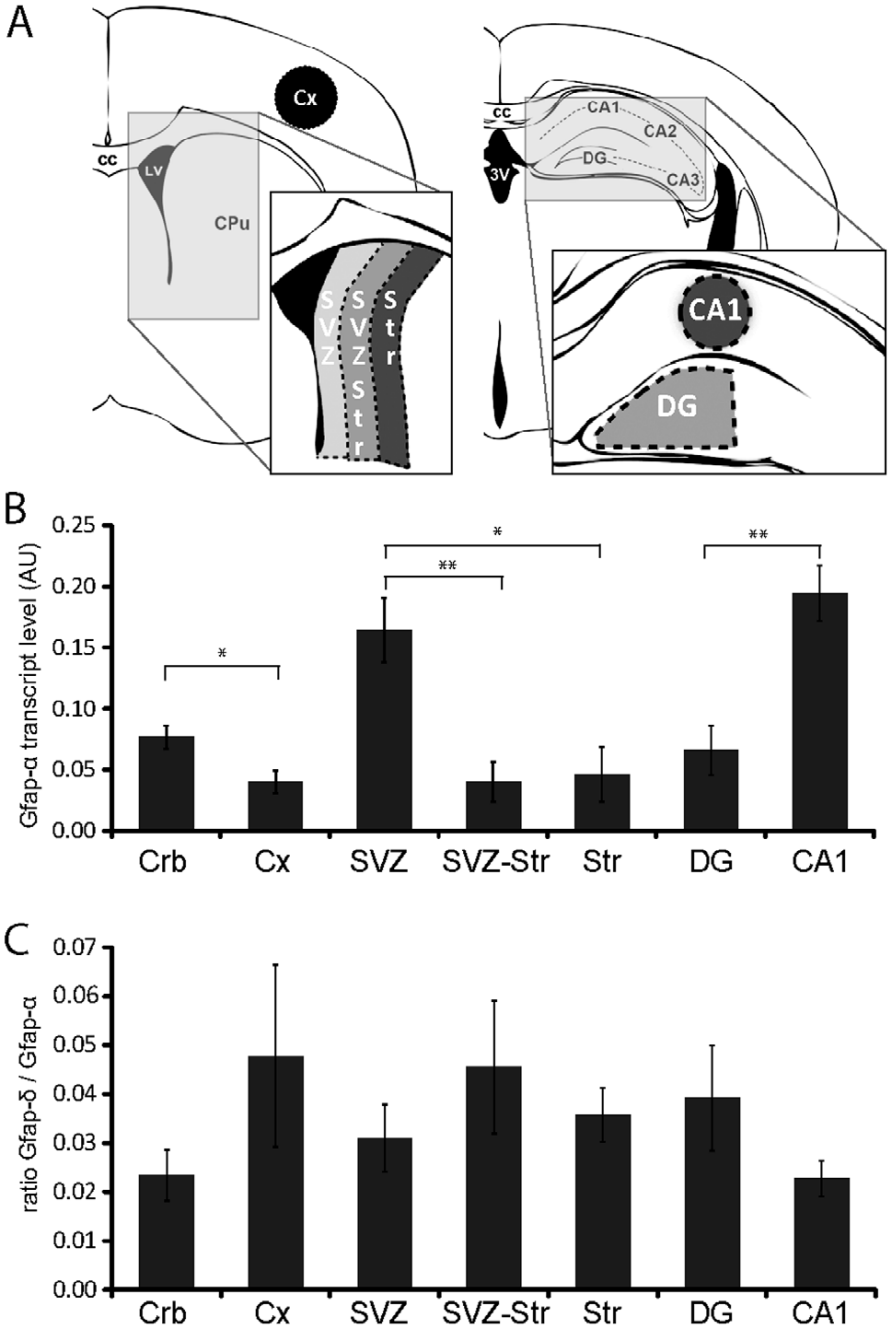
Transcript levels. (**A**) Schematic drawings of coronal sections to illustrate the areas isolated by LDM for RNA isolation. (**B**) Normalized transcript levels of Gfap-α in different brain areas in arbitrary units (AU). Gfap-α displays differential transcript levels between brain areas. Note the enhanced levels in the SVZ compared to the adjacent tissue. (**C**) Ratio of Gfap-δ/Gfap-α transcript levels shows no detectable differences between brain regions. Data is presented as mean ± SEM, n = 7. * P<0.05; ** P<0.01.

Gfap-α, Gfap-β, Gfap-γ, Gfap-δ, Gfap-κ, and Gfap-ζ were detected in all brain regions isolated from 8 week old WT mouse brains. Low Gfap-ΔEx7 transcript levels were occasionally observed and Gfap-Δ164 was never detected. Assays for Gfap-Δ135 and Gfap-ΔEx6 showed no cross-reactivity with Gfap-α, however both assays lack sensitivity and are not able to detect copy numbers lower than 0.003% of Gfap-α. Given this limitation, no evidence was found for Gfap-Δ135 and Gfap-ΔEx6 expression. After normalizing transcript levels to the geomean of the reference genes (Gapdh, Actb, Hprt, Rn18s) from the same samples, Gfap-α was found to be differentially expressed between regions (Figure 2A). The other isoforms followed this expression pattern and the ratio of the different isoforms over the Gfap-α levels revealed no differences between brain areas From this we conclude that Gfap-δ expression is not relatively higher in the neurogenic regions SVZ or dentate gyrus compared to the adjacent isolated tissue. In a second experiment, the lateral wall of the lateral ventricles was manually dissected and samples from striatum and cortex were taken. The determined Gfap-δ/Gfap-α transcript level ratio was not significantly different between SVZ samples and cortex (*P*  = 0.78; n = 9) or striatum (*P*  = 0.28). Transcript level for Ki67, a proliferation marker, was significantly higher in SVZ compared to cortex (15 fold increase; *P*<0.0004) and to striatum (11 fold increase; *P*<0.0003).

#### (ii) Gfap isoforms transcript levels at different ages in WT mouse cortex

Gfap-α, -β, -γ, -δ, -κ, and Gfap-ζ mRNA levels were determined in 51 mouse cortex cDNA samples of WT animals ranging in age from 3 to 18 months. Statistical analysis did not reveal any age-related changes. Gfap-Δ164 was not detected in any of the samples and no evidence was found for Gfap-Δ135 and Gfap-ΔEx6 expression. Using the plasmid calibration curves, the relative expression of the isoforms was estimated. Setting the amount of Gfap-α at 100%, Gfap-β was 0.076±0.012%, Gfap-γ 0.29±0.03%, Gfap-δ 7.9±0.3%, Gfap-κ 0.97±0.08% and Gfap-ζ 4.5±0.2% (n = 31; mean ± SEM).

#### (iii) Gfap isoform transcript levels in APPswePS1dE9 mouse cortex

**Figure 3:**
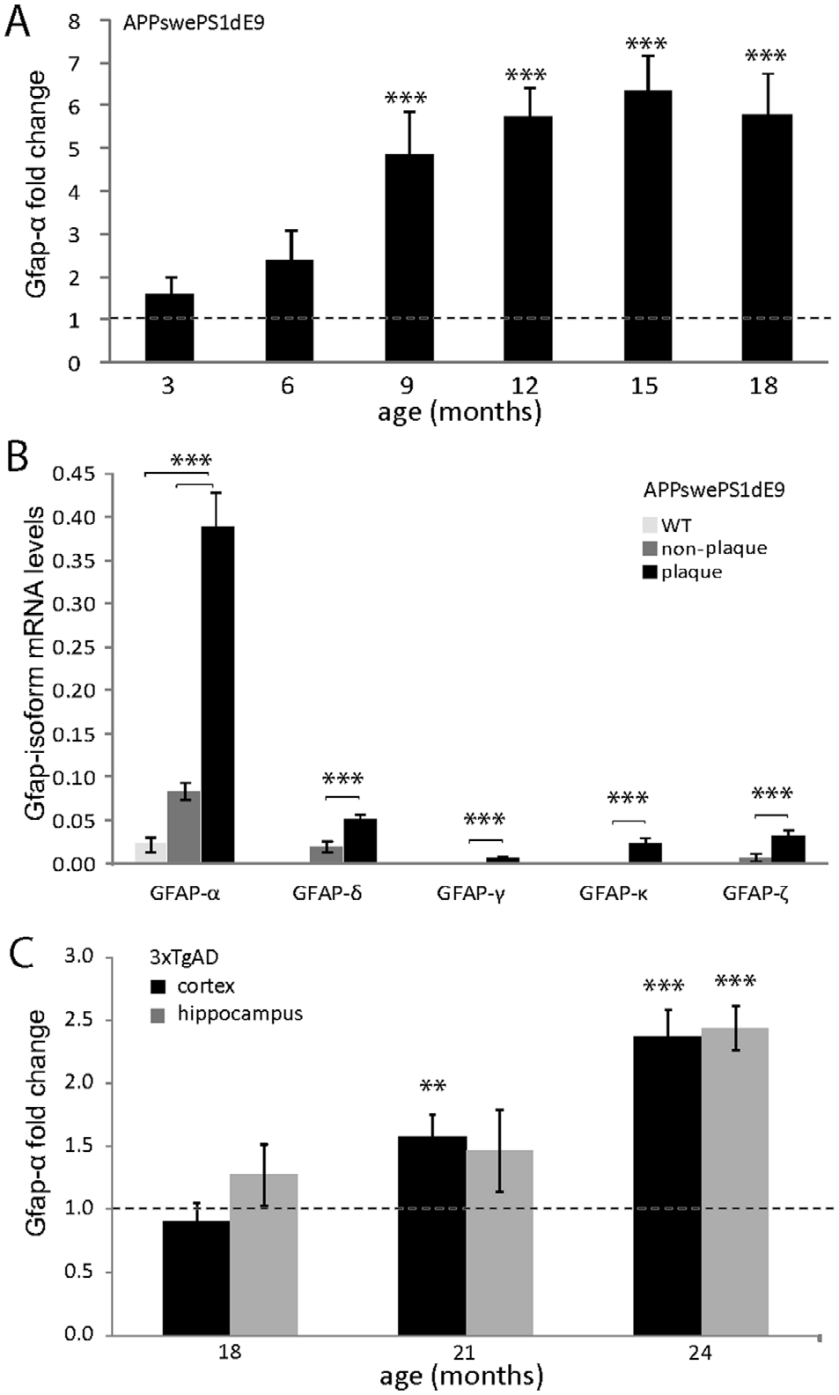
Transcript levels in aged APPswePS1dE9 and 3xTgAD mice. (**A**) Transcript levels of Gfap-α in cortex of APPswePS1dE9 mice increase with age (months). Data is presented as fold change ± SEM compared to age-matched WT animals (dashed line). Student’s t-test against age-matched WT mice. (**B**) Transcript levels of Gfap isoforms in LMPC isolated tissue samples from the cortex of 9 month old WT and APPswePS1dE9 mice (arbitrary units). In WT, Gfap-α and Gfap-δ could be detected but none of the other isoforms. In plaque samples from APPswePS1dE9, Gfap-α was detected at significantly higher levels compared to WT and to non-plaque samples. Gfap-δ, Gfap-γ, Gfap-κ, and Gfap-ζ were detectable in all AD plaque samples and in non-plaque samples at significantly lower levels. Data is presented as mean ± SEM, n = 7 for AD and n = 8 for WT. Statistics: Gfap-α WT vs. APPswePS1dE9: Mann-Whitney U-test and plaque-vs. non plaque: Wilcoxon matched pair signed rank test. (**C**) Transcript levels of Gfap-α in cortex and hippocampus of 3xTgAD mice increase with age. Data is presented as fold change ± SEM compared to age-matched WT animals (dashed line). Student’s T-test against age-matched WT mice. * P<0.05; ** P<0.01; *** P<0.001.

Compared to WT mice, statistically significant increased Gfap transcript levels were found in 9-month-old APPswePS1dE9 mice and older. Gfap-α levels were increased by 5.3 fold compared to their littermates (Figure 3A). Significantly elevated transcript levels with comparable fold changes were found for Gfap-β, -γ, -δ, -κ, and Gfap-ζ (Table 1). When examining the expression of the different isoforms relative to the Gfap-α levels in the same sample, we noted a small but significant difference in the ratio of Gfap-isoforms relative to Gfap-α between WT (n = 31) and APPswePS1dE9 mice (n = 42): for Gfap-β (from 0.076% in WT to 0.050% in APPswePS1dE9; *P*<0.004), Gfap-γ (0.29% to 0.21%; *P*<0.015), Gfap-δ (7.9% to 6.7%; *P*<0.011), Gfap-κ (0.97% to 0.59%; *P*<0.001), and Gfap-ζ (4.5% to 3.7%; *P*<0.007). Analysis of variance revealed no effect of age but a significant difference between APPswePS1dE9 and WT. The change in ratio indicates a small shift in the APPswePS1dE9 mice towards relatively more abundant levels of Gfap-α mRNA. For instance, compared to Gfap-δ or Gfap-ζ, the increase is 18 and 20%, respectively.

**Table 1:** Quantification of fold change in cortical transcript levels in APPswePS1dE9 mice compared age-matched WT.

	3 month	6 month	9 month	12 month	15 month	18 month	9–18 month
Gfap-α	1.58	2.39	4.88***	5.74***	6.33***	5.80***	5.27***
Gfap-β	0.70	2.29	1.95	3.44*	1.27	1.93	1.94**
Gfap-γ	0.97	2.22	2.05*	3.43***	6.51***	5.25***	4.06***
Gfap-δ	1.00	1.98	3.21***	4.75***	5.98***	4.35***	4.32***
Gfap-κ	0.84	2.80	2.29**	2.43**	7.31***	6.06***	6.07***
Gfap-ζ	0.84	2.39*	2.50**	4.33***	6.10***	3.02***	3.68***
Gfap-Δ135 1	n.d.	n.d.	n.d.	n.d.	n.d.	n.d.	n.d.
Gfap-Δ164	n.d.	n.d.	n.d.	n.d.	n.d.	n.d.	n.d.
Gfap-ΔEx6 1	n.d.	n.d.	n.d.	n.d.	n.d.	n.d.	n.d.
Gfap-ΔEx7 2	*n.s.*	*n.s.*	*P<0.031*	*n.s.*	*P<0.0005*	*P<0.028*	4.07***
Vim	1.07	1.33	1.38*	1.60***	2.21***	1.95**	1.72***
Nes	0.72	0.99	1.46	1.10	1.07	0.68	1.10
Synm H	1.17	0.98	1.32	1.11	1.11	0.90	1.07
Synm M	1.21	0.90	1.28	1.15	1.19	0.99	1.12
Aldh1l1	1.20	1.12	1.04	1.04	0.92	0.75	0.88
Glul	1.00	1.07	0.92	0.95	0.80	0.96	0.89
Glt1	1.03	0.63	1.44	1.34	1.16	1.14	1.27
Glast1	0.88	0.99	1.56	1.04	1.05	0.92	1.12
S100β	0.91	1.05	1.13	1.12	1.00	1.09	1.13
Fgfr3	1.09	1.05	1.11	1.02	1.28	0.74	1.00
Sox2	1.00	0.95	1.56	0.87	0.93	1.01	1.06

* *P*<0.05;

** *P*<0.01;

*** *P*<0.001;

Pooled data from 9–18 month old mice: n = 25 to AD; n = 38 to WT; n.d., not detectable.

1 Gfap-Δ135 and Gfap-ΔEx6 assays lack sensitivity to detect levels lower than 0.003% of the Gfap-α expression level.

2 Gfap-ΔEx7 was not detectable in all of the samples and fold change therefore fluctuates. The fold change is only presented for the pooled data from the 9–18 month groups; Mann-Whitney test was used for statistical analysis with non-detectable level set at 0; n.s., non significant.

Data are presented as fold change of **APPswePS1dE9** mice compared to expression levels found in WT mice of the same age.

Low expression levels of a new isoform, Gfap- were detected in 16 out of 53 (30%) WT mice and in 26/42 (62%) in APPswePS1dE9 mice. In the group of 9–18 month old APPswePS1dE9 mice this was 19/25 (76%) vs. 11/38 (29%) in WT. When detected, Gfap-ΔEx7 levels were 0.0033±0.0006% compared to Gfap-α. Vimentin transcript levels were also increased in APPswePS1dE9 mice, but the increase started later than that of Gfap, and was less prominent with a maximum 2 fold change at 15–18 months. Remarkably, mRNA levels of the intermediate filaments nestin (Nes), synemin-L (Symn-L) and synemin-H (Symn-H) [41] were not elevated as were several other astrocyte specific genes [42], such as glutamine synthetase (Glul) and glutamate transporter 1 (Glt1) (Table 1).

From APPswePS1dE9 mice, plaques and their immediate surroundings were collected by means of LMPC. For comparison, cortical areas of similar size were isolated from areas without plaques in APPswePS1dE9 and from WT mice at the age of 6 and 9 months. Plaque containing samples had significantly higher transcript levels of Gfap-α at 6 months (11.3±1.4 fold; *P*<0.0001) and at 9 months (17.4±1.8 fold; *P*<0.001; Figure 3B) compared to WT samples. Gfap-δ, Gfap-γ, Gfap-κ, and Gfap-ζ were not found in WT samples but were detectable in all APPswePS1dE9 plaque samples with an expression profile comparable to that observed in whole cortex cDNA samples. Transcript levels in non-plaque samples were significantly lower. Plaque containing samples showed a significant increase of vimentin transcript compared to WT (5.5±1.3 fold; *P*<0.002), no change of Synm-H and -M, while levels of nestin were not detectable. Transcript levels of the astrocyte specific genes Aldh1l1, Glul, Glt1, Glast1, Fgfr3, and Sox2 were not changed in either plaque or non-plaque containing samples compared to WT [38].

#### (iv) Gfap isoform transcript levels in 3xTgAD mouse cortex

Gfap isoform transcript levels were determined in 3xTgAD mice in cortex and hippocampus at the age of 18, 21, and 24 months. In line with the observation that most 3xTgAD mice start to develop plaques at 21 and 24 months, transcript levels of Gfap isoforms were increased approximately 2-fold at 24 month in both hippocampus and cortex (Table 2; Figure 3C). Gfap-Δ135, Gfap-Δ164 and Gfap-ΔEx6 were not detected in any of the samples. At 18 and 21 months, about half of the samples showed low levels of Gfap-ΔEx7. At 24 months, most of the samples, WT and 3xTgAD, had detectable Gfap-ΔEx7 levels (Table 2) without a significant increase in the 3xTgAD mice. The ratio of the different isoforms relative Gfap-α was in the cortex: Gfap-β 0.076±0.006%, Gfap-γ 0.25±0.004%, Gfap-δ 9.5±1.0%, Gfap-κ 1.2±0.2% and Gfap-ζ 4.8±0.4% (n = 8), and in the hippocampus: Gfap-β 0.062±0.007%, Gfap-γ 0.30±0.03%, Gfap-δ 8.6±03%, Gfap-κ 0.71±0.05% and Gfap-ζ 5.4±0.2% (n = 16). The relative expression profile was not significantly different between cortex and hippocampus. In the 3xTgAD compared to WT mice, a small but significant decrease in the ratio of Gfap-isoforms relative to Gfap-α was observed in cortex and hippocampus. This was found all ages and, as in the APPswePS1dE9 mice, this indicates a somewhat higher expression of Gfap-α of 10–20%.

**Table 2:** Quantification of fold change in hippocampal and cortical transcript levels in 3xTgAD mice compared age-matched WT.

	18 month	21 month	24 month	18 month	21 month	24 month
	HPC	HPC	HPC	CX	CX	CX
	TG n = 6	TG n = 6	TG n = 11	TG n = 6	TG n = 6	TG n = 11
	WT n = 4	WT n = 8	WT n = 4	WT n = 8	WT n = 8	WT n = 8
Gfap-α	1.27	1.47	2.44***	0.90	1.57*	2.37***
Gfap-β	1.15	1.05	2.03*	0.80	1.53	2.30***
Gfap-γ	1.07	1.41	2.46***	1.28	1.83*	2.44***
Gfap-δ	0.98	1.44*	2.10***	0.80	1.13	1.80***
Gfap-κ	1.00	1.38	2.57***	0.82	1.23	1.57**
Gfap-ζ	0.98	1.46	2.15***	0.94	1.33**	2.08***
Gfap-Δ135^1^	n.d.	n.d.	n.d.	n.d.	n.d.	n.d.
Gfap-Δ164	n.d.	n.d.	n.d.	n.d.	n.d.	n.d.
Gfap-ΔEx6^1^	n.d.	n.d.	n.d.	n.d.	n.d.	n.d.
Gfap-ΔEx7^2^	4/10	7/14	14/15; n.s.	4/8	5/10	10/13; n.s.

* *P*<0.05;

** *P*<0.01;

*** *P*<0.001;

Pooled data from 9–18 month old mice: n = 25 to AD; n = 38 to WT; n.d., not detectable. HPC, hippocampus; CX, cortex.

1 Gfap-Δ135 and Gfap-dEx6 assays lack sensitivity to detect levels lower than 0.003% of Gfap-α.

2 Gfap-ΔEx7 was not detectable in all of the samples; indicated number of mice (TG+WT) with detectable signal over all mice studied. No change in AD vs WT was detected.

Data are presented as fold change of **3xTgAD** mice compared to expression levels found in WT mice of the same age.

### Specificity of Antibodies Against Mouse GFAPδ, GFAPκ, and GFAP^+1^ (GFAPΔ164, GFAPΔEx6, GFAPΔEx7)

**Figure 4:**
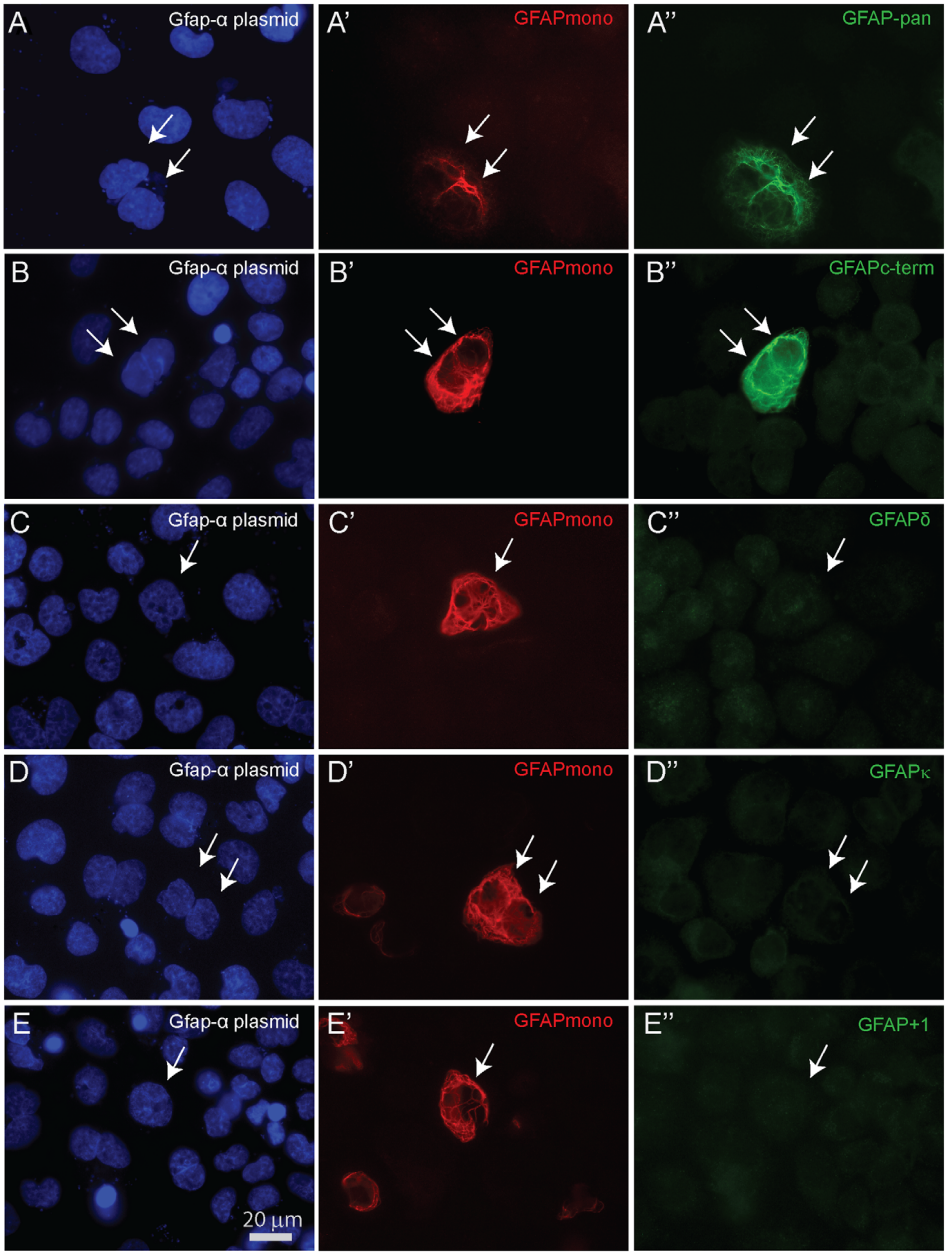
GFAPα transfected cells stained with various GFAP antibodies. All panels show SW13/cl.2 cells transfected with full length msGfap-α, stained with GFAP monoclonal antibody to detect successfully transfected cells and double stained with the different polyclonals. (**A–A’**) GFAPpan and (**B–B’**) GFAPc-term antisera are able to detect GFAPα composed IF networks, whereas (**C–C’**) msGFAPδ, (**D–D’**) msGFAPκ, and (**E–E’**) msGFAP+1 display no reactivity against the canonical GFAPα. Panels **A–E** show DAPI staining, a fluorescent stain that binds to DNA.

**Figure 5:**
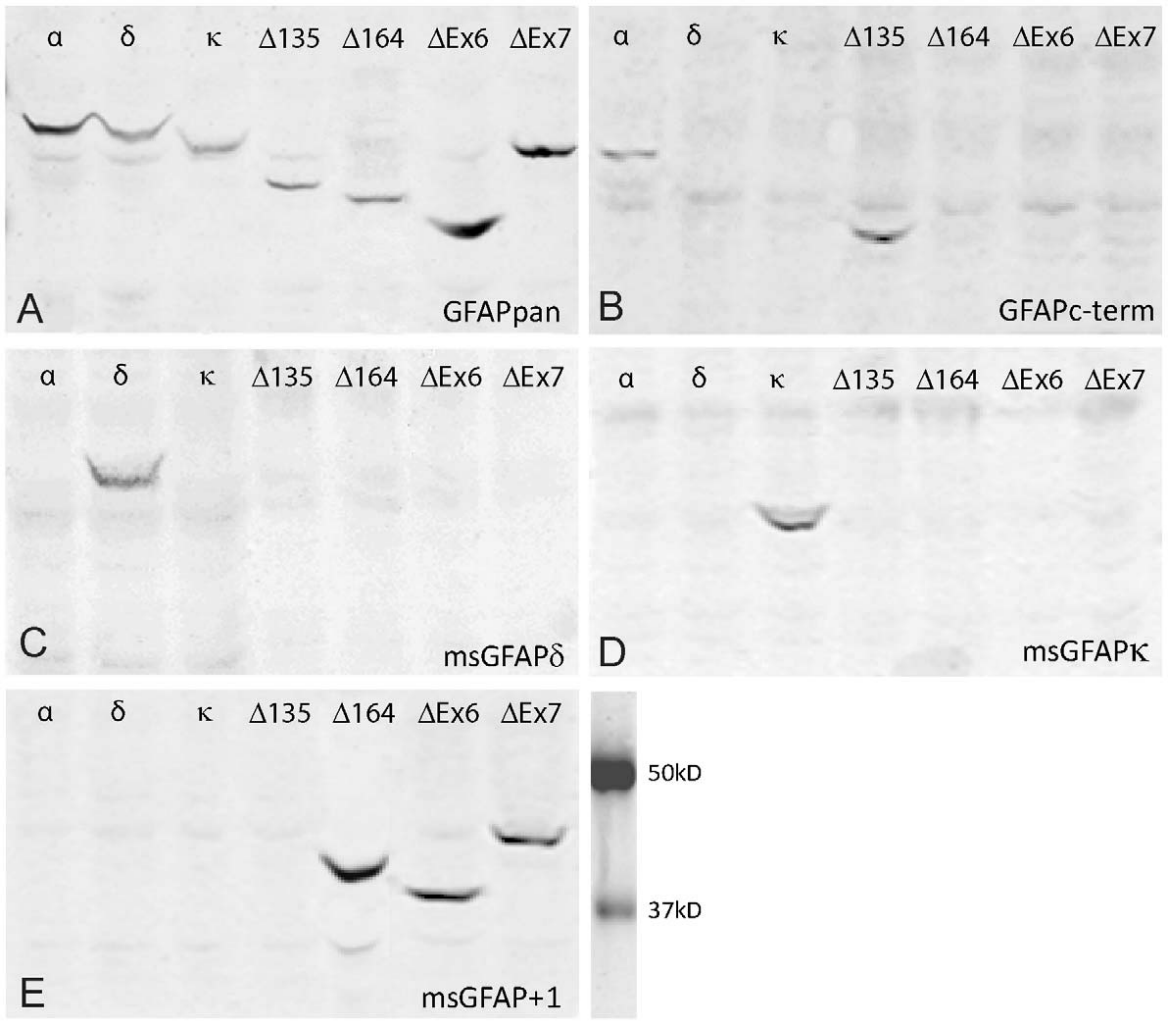
Western blots on transfected SW13/cl.2 cells. Protein samples prepared from SW13/cl.2 cells transfected with 7 different GFAP-isoforms, indicated at the top of each lane, were blotted. Blots were incubated with GFAP isoform-specific antibodies indicated at the bottom of each panel: (**A**) GFAPpan, detecting all isoforms. Note the small difference in molecular weight of GFAPα and GFAPδ in the transfected cells. (**B**) GFAPc-term, detecting the C-terminal sequence encoded by exon 9 only present in GFAPα and GFAPΔ135, (**C**) msGFAPδ, showing specificity for GFAPδ, (**D**) msGFAPκ, showing specificity for GFAPκ, and (**E**) msGFAP+1 antibody able to detect the GFAP isoforms typified by the +1 shifted reading frame in GFAPΔ164, GFAPΔEx6, and GFAPΔEx7.

Antibodies directed against mouse GFAP isoforms (Table 3) were tested on GFAPα transfected SW13/cl.2 cells. GFAPpan, GFAPmono, GFAPc-term detected the full length GFAPα protein (Figure 4A–A”, B-B”). While our msGFAPδ, msGFAPκ, and msGFAP^+1^ antibodies showed no detectable cross-reactivity with GFAPα-positive IF networks (Figure 4C–C”, D–D”, E–E”). Additional specificity studies were performed on western blots from SW13/cl.2 cells transfected with different isoforms. The commonly used rabbit polyclonal Z0334 (Dako; GFAPpan) was able to detect a single band with all 7 isoforms tested. Full length sequence information is not available for GFAPβ, GFAPγ, and GFAPζ, thus these isoforms were not included. The antibodies showed the expected detection patterns (Figure 5, Table 4). Furthermore, SW13/cl.2 cells were transfected with each isoform and then stained by GFAPpan to detect transfected cells. These cells were also double stained with the panel of other antibodies. The resulting specificity pattern corroborated the western blot results as shown in Table 4.

**Table 3:** Table **3.** Mouse GFAP-isoform specific antibodies and other antibodies used.

Name	Specificity	Epitope	Source
GFAPpan	All isoforms	Full length GFAP cow	Dako - rabbit polyclonal Z0334 (1∶2000)
GFAPmono	α, δ, κ, Δ135	Pig (ITIPVQTFSNLQIR)	Sigma - mouse monoclonal G-A-5 (1∶6000)
GFAPc-term	α, Δ135	Human GFAP C19	SantaCruz - goat polyclonal Sc6170 (1∶400)
		EMRDGEVIKESKQEHKDVM	(EMRDGEVIKDSKQEHKDVVM of msGFAP)
msGFAPδ	δ	Q*E*IENGALPALP ∼TG *	NIN rabbit polyclonal (bleeding: 15-12-2003; 1∶500)
msGFAPκ	κ	EIQVLLESLRDPPRRS∼KLH**	NIN rabbit polyclonal (bleeding: 29-04-2008; 1∶500)
msGFAPκ	κ	EIQVLLESLRDPPRRS∼TT ***	NIN rabbit polyclonal (bleeding: 30-03-2009; 1∶500)
msGFAPκ	κ	ESLRDPPRRS∼TT	NIN rabbit polyclonal (bleeding: 30-03-2009; 1∶500)
msGFAP^+1^	ΔEx6, Δ164, ΔEx7	RGKDCGDAGW∼TGl	NIN rabbit polyclonal (bleeding: 29-04-2008; ip; 1∶500)
Vimentin		Recombinant vimentin	Millipore - chicken polyclonal AB5733 (1∶1500)
Nestin		Mouse nestin-peptide mix	Novus Biologicals - chicken polyclonal NB100-1604 (1∶1000)
Synemin		C-terminal domain of human α- and β-synemin	MUbio MUB1704P - goat polyclonal (1∶1000)
Aβ		Epitope Aβ3–8 (6E10)	Signet-Covance (1∶15,000)
BrdU			AbD Serotec (1∶2500)
Ki67			Novacastra (1∶2500)
Actin			Hybridoma Bank - mouse monoclonal JLA20 (1∶2000)

* TGl  =  thyroglobulin; note the erroneous insertion of *E* when designing the epitope sequence.

** Keyhole limpet hemocyanin.

*** Tetanus Toxoid.

ip, immunopurified.

**Table 4:** Specificity of antibodies directed against mouse GFAP isoforms tested on SW13/cl.2 cells transfected with mouse full length GFAP isoform cDNAs.

Transfection	GFAPpan	GFAPmono	GFAPc-term	msGFAPδ	msGFAPκ	msGFAP^+1^
	Rabbit	Mouse	Goat	Rabbit	Rabbit	Rabbit
	1∶6,000	1∶3,000	1∶3,000	1∶3,000	1∶3,000	1∶6,000
GFAP-α	+	+	+	−	−	−
GFAP-δ*	+	+	−	+	−	−
GFAP-κ	+	+	−	−	+	−
GFAP-Δ135	+	+	−**	−	−	−
GFAP-Δ164	+	−	−	−	−	+
GFAP-ΔEx6	+	−	−	−	−	+
GFAP-ΔEx7	+	−	−	−	−	+

* Transfection with human GFAPδ showed panGFAP positive cells that were negative for msGFAPδ.

** GFAPd135 is expected to have a conventional GFAPα C-terminus and the observed absence of staining.

**Figure 6:**
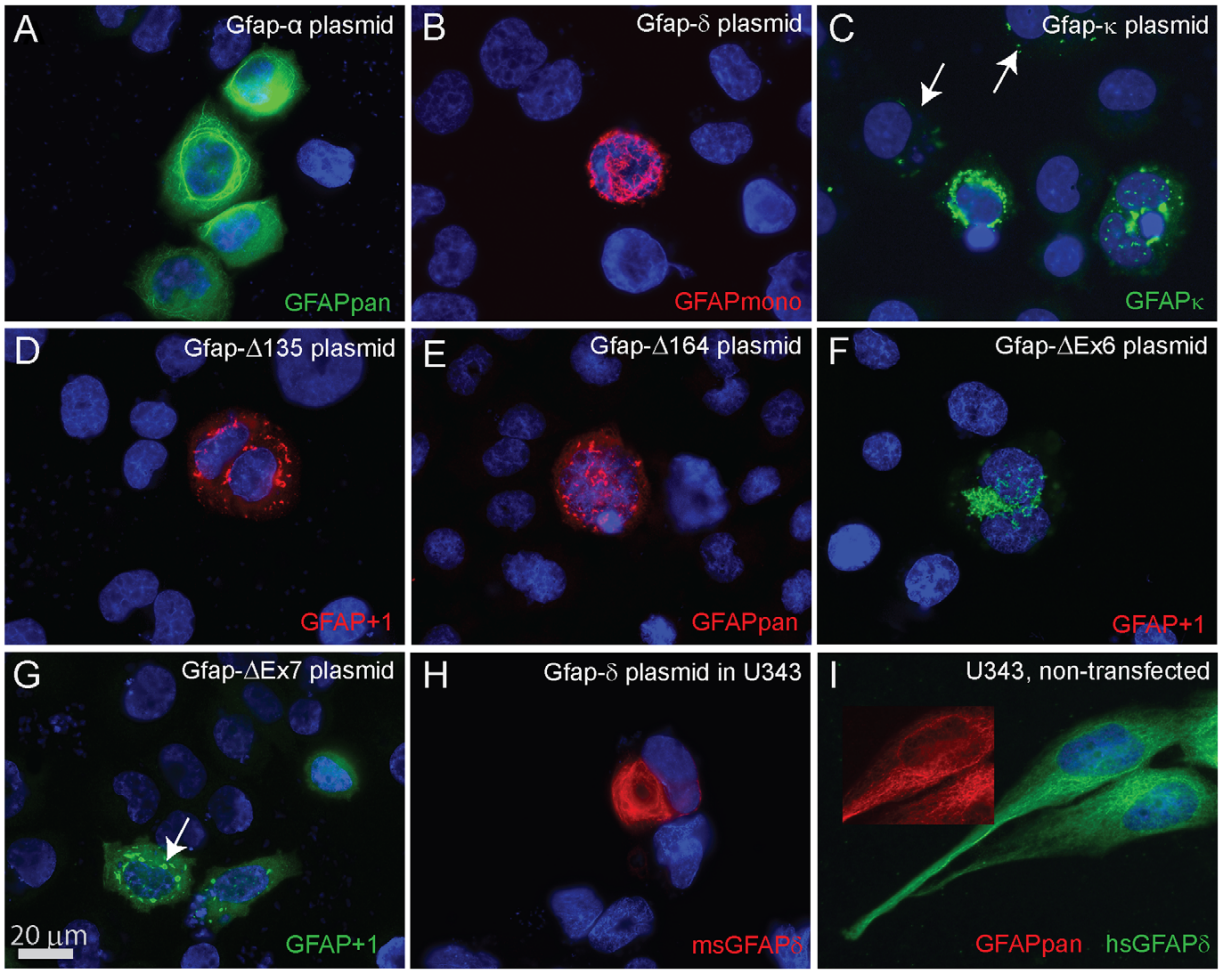
Expression of GFAP isoforms results in different IF network morphologies. Expression of GFAP isoforms in SW13/cl.2 cells. Transfected cultures were double stained with GFAPmono in order to identify successfully transfected cells and with the different GFAP antibodies listed in Table 3. The selection shown here illustrates that GFAPα is the only isoform yielding a network composed of long filaments (**A**), whereas GFAPδ (**B**), GFAPκ (**C**), GFAPΔ135 (**D**), GFAPΔ164 (**E**), GFAPΔex6 (**F**), and GFAPΔEx7 (**G**) lead to aberrant networks. When msGFAPδ was overexpressed in U343, a human astrocytoma cell line with an endogenous IF network, the resulting msGFAPδ network showed longer filaments (**H**) (compare with 4B), but this network was different from the much weaker endogenous network of GFAP composed of GFAPα and GFAPδ observed in non-transfected cells recorded at longer exposure time (**I**).

The isoforms generated GFAP-positive networks SW13/cl.2 cells with different morphologies (Figure 6A–G). GFAPα generated an extensive diffuse network composed of fine long filaments (Figure 6A). GFAPδ led to a more condensed network of short thick filaments (Figure 6B). GFAPκ resulted in a similar type of network as seen with GFAPδ within some cells but also a more punctate pattern in others (Figure 6C). The GFAPΔ135 network was composed of short thick filaments (Figure 6D). GFAPΔ164 and GFAPΔEx6 have a punctate appearance and were condensed into one or more spots in several cells (Figure 6E,F)). GFAPΔEx7 has a diffuse expression throughout the cell with some intensely labeled large-sized spots (Figure 6G). When cells with a pre-existing GFAP network, the U343 cells, were transfected, the formed network had a less aberrant structure (Figure 6H). However, the IF network still differed from the endogenous network in non-transfected cells (Figure 6I).

**Figure 7:**
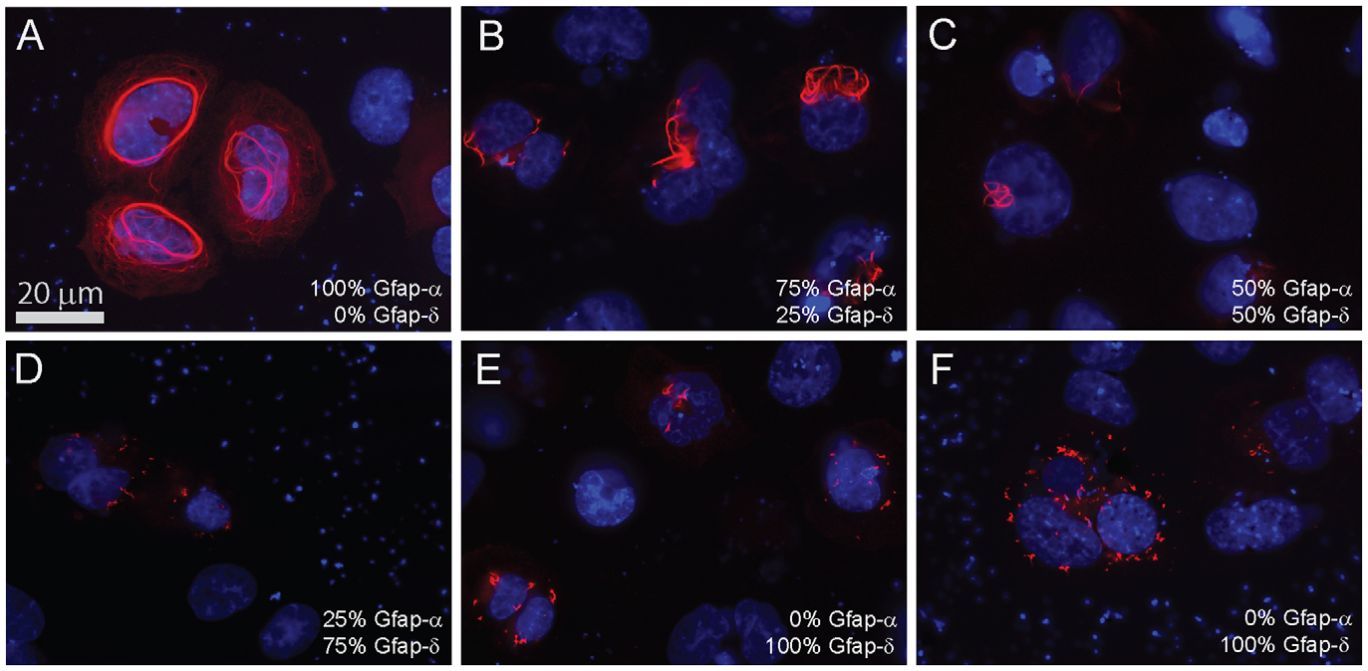
Co-expression of GFAPα and GFAPδ at different ratios results in different IF network morphologies. Co-transfection of SW13/cl.2 cells with different ratios of GFAPα and GFAPδ encoding vectors. Transfected cultures fixed 24 h after transfection and stained with GFAPpan to study the morphology of the resulting IF networks. Transfection of GFAPα without GFAPδ yielded complex networks composed of long filaments present throughout the cell (**A**), whereas 75% GFAPα/25% GFAPδ results in condensed networks (**B**). A 50% GFAPα/50% GFAPδ ratio yields small networks or just isolated short filaments (**C**). At 25% GFAPα/75% GFAPδ and 100% GFAPδ only short “squiggles” were observed (**D–F**).

When co-transfecting different ratios of Gfap-α/−δ in SW13/cl.2 cells, only Gfap-α transfections resulted in networks with long filaments (Figure 7A). At a ratio of 75% Gfap-α and 25% -δ, networks were less well developed and did not occupy the whole cell (Figure 7B). At a ratio of 50% Gfap-α and 50% -δ, condensation was stronger (Figure 7C). A punctate pattern of staining throughout the cell was found at 25% Gfap-α and 75% -δ (Figure 7D) and at 100% Gfap-δ (Figure 7E–F).

### Western Blots on Protein Samples of APPswePS1dE9 and WT Cortex

**Figure 8:**
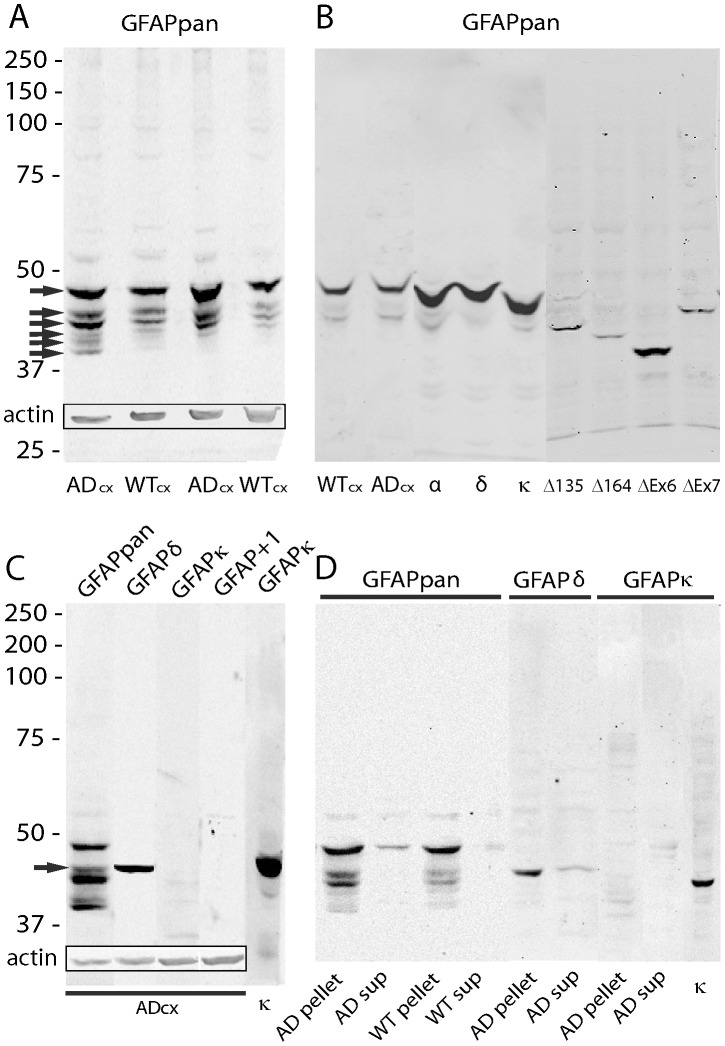
Western blots of mouse cortex WT and AD 12–15 month cortex samples. (**A**) Protein samples (30 µg/lane) from cortex of two pairs of AD and WT mice aged 15 months (left lanes) and 9 month old (right lanes). Blot was probed with the GFAPpan antibody (1∶6000) revealing several bands (arrows). As check for comparable loading of the lanes, blots were also probed for actin (rectangular insert). (**B**) Protein samples from cortex of 15 month WT and AD (15 µg/lane) and lysates of cells transfected with the different isoforms. Blots were probed with the GFAPpan antibody (1∶6000). Alignment of the cell lysates with the cortex samples does not yield clear identification of the multiple bands detected in the cortex. (**C**) Identical protein samples (15 µg/lane) of a 9 month old AD mouse were run in adjacent lanes and probed with GFAPpan (1∶6000), GFAPδ (1∶2000), GFAPκ (1∶500), and GFAP+1 (1∶500). In line with the immunohistochemical data only GFAPδ detected a single band corresponding with the slightly smaller-sized band than the dominant GFAPpan band around 48 kDa (arrow). Insert shows detection of actin in the same lanes. As positive control for GFAPκ, a lysate of GFAPκ transfected cells was run in parallel. (**D)** Protein samples (15 µg/lane) from pellet and supernatant (sup) fractions were run and blotted and probed with GFAPpan (1∶6000), GFAPδ (1∶2000), and GFAPκ (1∶500). For both AD and WT, most GFAP is present in the pellet fraction with a small amount located in the soluble fraction typically only the largest of the GFAPpan bands. GFAPδ is present in the pellet and GFAPκ was not detected as was GFAP+1 (not shown). As positive control for GFAPκ, a lysate of GFAPκ transfected cells was run in parallel.

Detection of GFAP on western blots of protein samples from 12 and 15 month old WT and APPswePS1dE9 mice with the GFAPpan antibody revealed, at high protein load, two strong and four weaker bands without a clear difference in pattern between WT and APPswePS1dE9. However, the intensity of the APPswePS1dE9 samples was consistently higher (Figure 8A). Co-blotting of mouse cortex homogenates together with the homogenates of the transfected cells did not allow an unambiguous identification of the different GFAP-isoforms in cortex samples (Figure 8B). Applying our panel of isoform specific GFAP antibodies on blots of WT and APPswePS1dE9 cortex showed a single band for GFAPδ, and no detectable staining for GFAPκ or GFAP^+1^ (Figure 8C; cell lysates of transfected cells were used as positive controls). The GFAPδ band size corresponded with one of the GFAPpan detected bands (Figure 8C). In supernatant protein fractions only GFAPpan and GFAPδ gave a weak signal and no differences between APPswePS1dE9 and WT were noted (Figure 8D).

### GFAPα and GFAPδ Immunostaining in WT Mouse Brain

**Figure 9:**
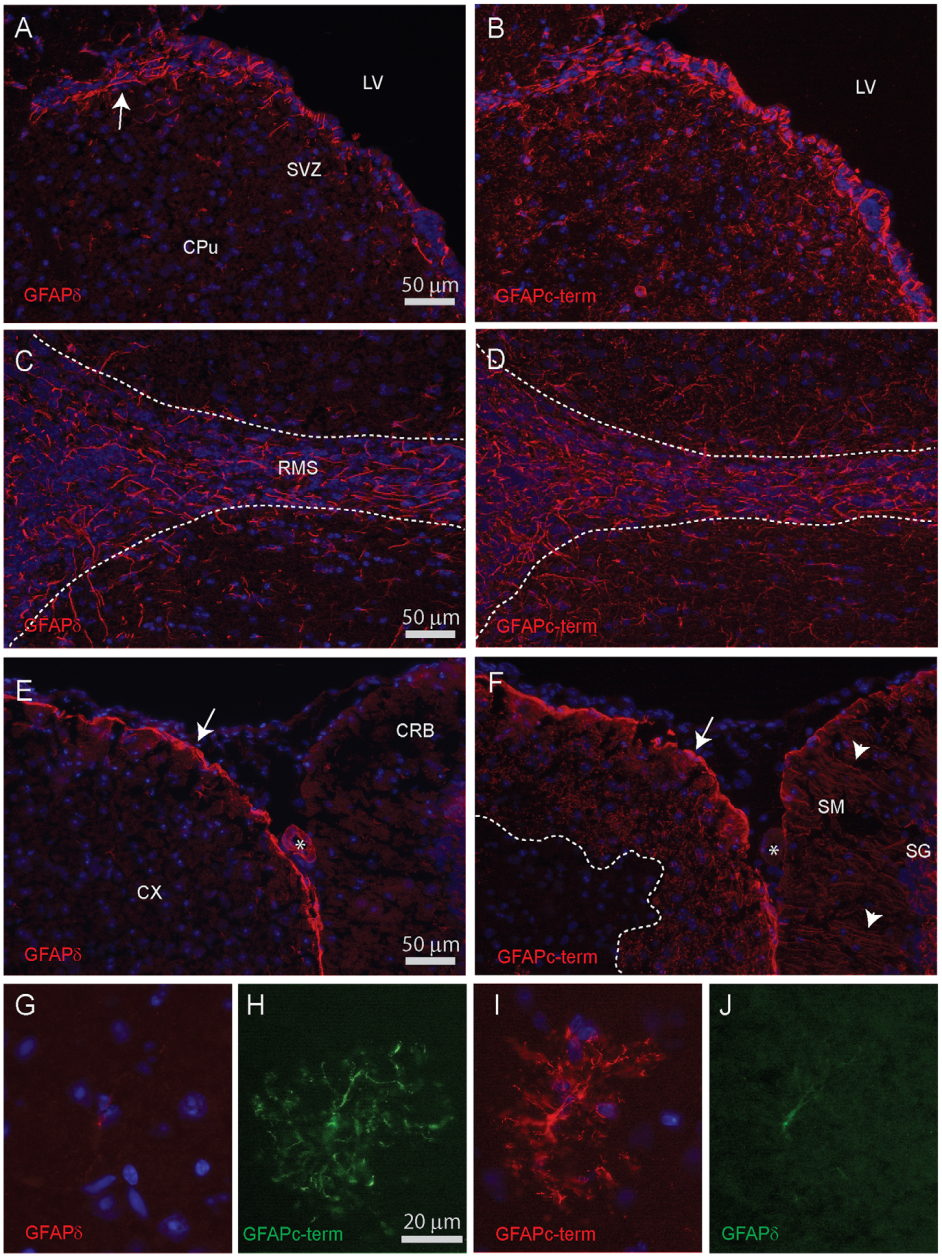
GFAPα and GFAPδ stainings in mouse brain. Two adjacent sagittal sections were both incubated with the same mix of GFAPc-term (raised in goat) and GFAPδ (raised in rabbit) antibodies. Thereafter one section was incubated with donkey-anti-goat-Cy3 and donkey-anti-rabbit-DL488-Cy3. The other section with donkey-anti-goat-DL488 and donkey-anti-rabbit-Cy3. Shown are photomicrographs recorded from the Cy3 channel in both sections, all recorded and processed at identical settings. This avoids any bias caused by the different sensitivities of detection by either Cy3 or DL488 fluorophores. The 6 µm thick sections were cut from frozen brains and after mounting on glass slide shortly fixed with PFA. This procedure was optimal for both antibodies. (**A,B**) Lateral ventricle (LV), SVZ and caudate putamen (CPu). Arrow indicates the start of the RMS towards the olfactory bulb at the left. Both GFAPc-term the localization of GFAPδ in processes in the SVZ while GFAPc-term is expressed in the SVZ and in fine processes in CPu. Visualization in the DL488 channel confirms this pattern. (**C,D**) Sections showing the end of the RMS near the olfactory bulb. GFAPδ and GFAPc-term stain processes in the RMS with the surrounding parenchyma is mostly GFAPc-term. (**E,F**) GFAPδ is highly expressed in astrocytes near the pial surface of cortex (CX; arrow). In cerebellum (CRB) no GFAPδ staining is observed. A large arterial blood vessel stains for GFAPδ (asterisk). GFAPc-term labels glial processes in stratum moleculare (SM) and stratum granulosum (SG; small arrows). The subpial zone in the cortex is GFAPc-term positive but GFAPδ negative. (**G,H**) Double staining of a GFAP-positive single protoplasmic astrocyte in the cortex to illustrate the near absence of GFAPδ. The same cell was observed in the adjacent section with reversed secondary antibodies (**I,J**).

Immunostainings of mouse brain sections with the panel of antibodies (Table 3) yielded staining patterns for GFAPpan, GFAPmono, GFAPc-term and GFAPδ. Double staining with these antibodies showed overlapping distributions in mouse brain. No specific staining pattern was obtained with the different antisera against GFAPκ and GFAP^+1^, which is in line with the western blot data. Because of the absence of detectable GFAPΔ135 transcript level, we assume that the GFAPc-term pattern is in fact GFAPα-specific. Double stainings with GFAPc-term and GFAPδ antibodies were therefore used to compare the distribution of GFAPα and GFAPδ in mouse brain by double staining and comparing staining pattern and intensity in adjacent sections with images recorded at identical settings (Figure 9). GFAPα and GFAPδ expression overlapped in all sections studied. However, GFAPδ had a more restricted pattern and, when detected, staining intensity was generally lower compared to that of GFAPα. No or just sparse GFAPδ immunostaining was found in caudate putamen (Figure 9A,B), molecular and granular layer of the cerebellum (Figure 9E,F), and in GFAPα-positive protoplasmic astrocytes in the cortex and hypothalamus (Figure 9E–J). Weak GFAPδ immunostaining was detectable in hippocampal astrocytes, with a staining confined to the primary processes of the cells. Intense GFAPδ immunostaining was found in astrocytes lining the pial surface (Figure 9E,F), and in astrocytes in the SVZ (Figure 9A,B), and RMS (Figure 9C,D).

**Figure 10:**
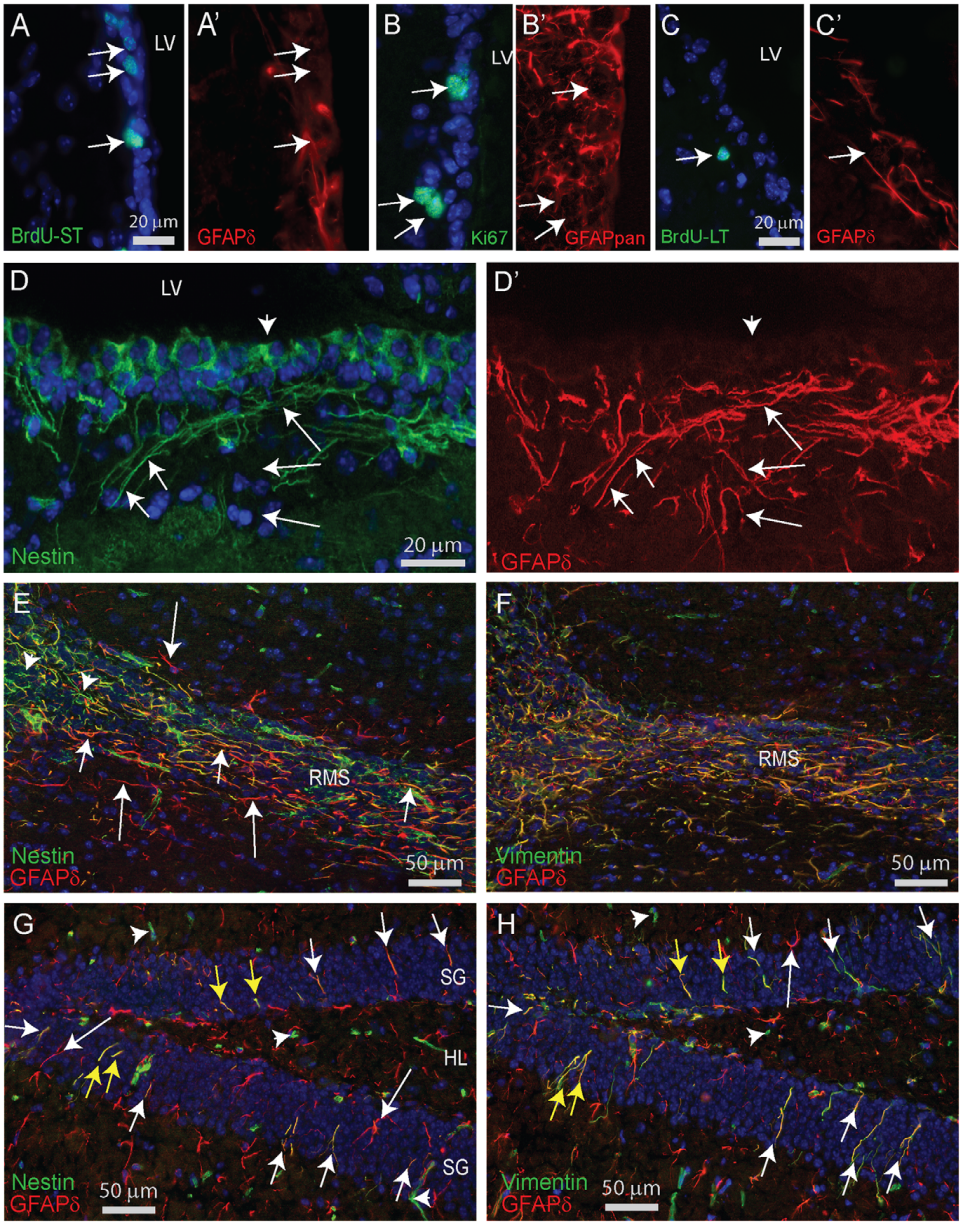
Immunocytochemical stainings SVZ. (**A,A’**) Staining for BrdU with short-term survival after the last BrdU injection reveals proliferating cells (arrows) in the SVZ of a 6 month old mouse. Assigning GFAPδ staining to a specific BrdU-labelled nucleus is not feasible. (**B,B’**) Double staining with GFAPpan and Ki67 as a marker for proliferating cells in the SVZ. (**C,C’**) Staining for BrdU after long-term survival after the last BrdU injection reveals only a few BrdU-positive cells in the SVZ. These cells represent the slowly dividing stem cell pool residing in the SVZ. No clear expression level of GFAPδ could be assigned to these cells. (**D,D’**) Double staining of nestin and GFAPδ demonstrates a clear co-localization of GFAPδ positive processes with nestin in the SVZ (short arrows) but deeper in the parenchyma GFAPδ processes are nestin negative (long arrows). Nestin is also localized in the ependymal cell layer (arrow head). (**E**) A section containing the RMS stained for GFAPδ and nestin. The olfactory bulb is located at the left. Note the prominent staining of long GFAPδ positive processes (arrows), most of which are also nestin-positive (short arrows) while those located more at the RMS border are nestin-negative (long arrows). Towards the olfactory bulb nestin-only processes becomes more prominent (arrow heads). (**F**) The adjacent section of the one shown in E but stained for GFAPδ and vimentin. Note the complete overlap of both patterns. (**G**) The hippocampal dentate region stained for GFAPδ and nestin. Short arrows indicate GFAPδ and nestin double positive fibers stretching into the granular cell layer typical for the SGZ stem cells. The cell bodies of these cells could not be identified. Long arrows indicate GFAPδ positive, nestin negative representing conventional astrocytes. Arrowheads point at nestin positive blood vessels. (**H**) The adjacent section of the one shown in G but stained for GFAPδ and vimentin. Short arrows indicate GFAPδ and vimentin double positive fibers stretching into the granular cell layer typical for the SGZ stem cells. Careful alignment on blood vessels identified process belonging to the same cells; indicated by yellow arrows demonstrating that GFAPδ, nestin, and vimentin are expressed by the same cells. RMS, rostral migratory stream; SG stratum granulosum; HL, hilus.

Since GFAPδ in the human brain has a preferential expression in proliferating astrocytes of the SVZ, we studied the SVZ of mice in more detail. We performed co-stainings of GFAPδ and BrdU in mice that were sacrificed at either short-term or long-term survival after BrdU injections to label proliferating cells and quiescent stem cells, respectively. Although proliferating cells are surrounded by GFAPδ-positive processes, we were not able to assign GFAPδ-positive filaments to specific BrdU-positive nuclei (Figure 10A,A' and 10C,C'). The combination of GFAPpan or GFAPδ with Ki67 also did not establish an unambiguous GFAP localization in proliferating cells (Figure 10B,B'). Double staining for GFAPδ and nestin revealed that all GFAPδ processes near the ventricular wall are nestin positive but deeper in the parenchyma the GFAPδ processes are nestin negative (Figure 10D,D'). At the origin of the RMS near the SVZ, all GFAPδ processes are nestin positive but along the RMS toward the olfactory bulb the two staining patterns dissociate gradually (Figure 10E). In SVZ and along the RMS, all GFAPδ processes are vimentin positive (Figure 10F). In the SGZ of the hippocampal dentate region, fine nestin positive processes were found to cross the granular cell layer and to stretch in to the molecular layer. These processes belong to type 1 radial astrocytes, the astroglial stem cells in the SGZ [43]. All nestin positive processes were GFAPδ positive (Figure 10G). Such GFAPδ processes were also found to be double positive for GFAPα and vimentin (Figure 10H). Horizontally oriented processes of the horizontal glia in the SGZ were nestin negative but vimentin and GFAPδ positive [43].

### GFAP Immunostaining in Cortex of WT and Around Plaques in AD Mice

**Figure 11:**
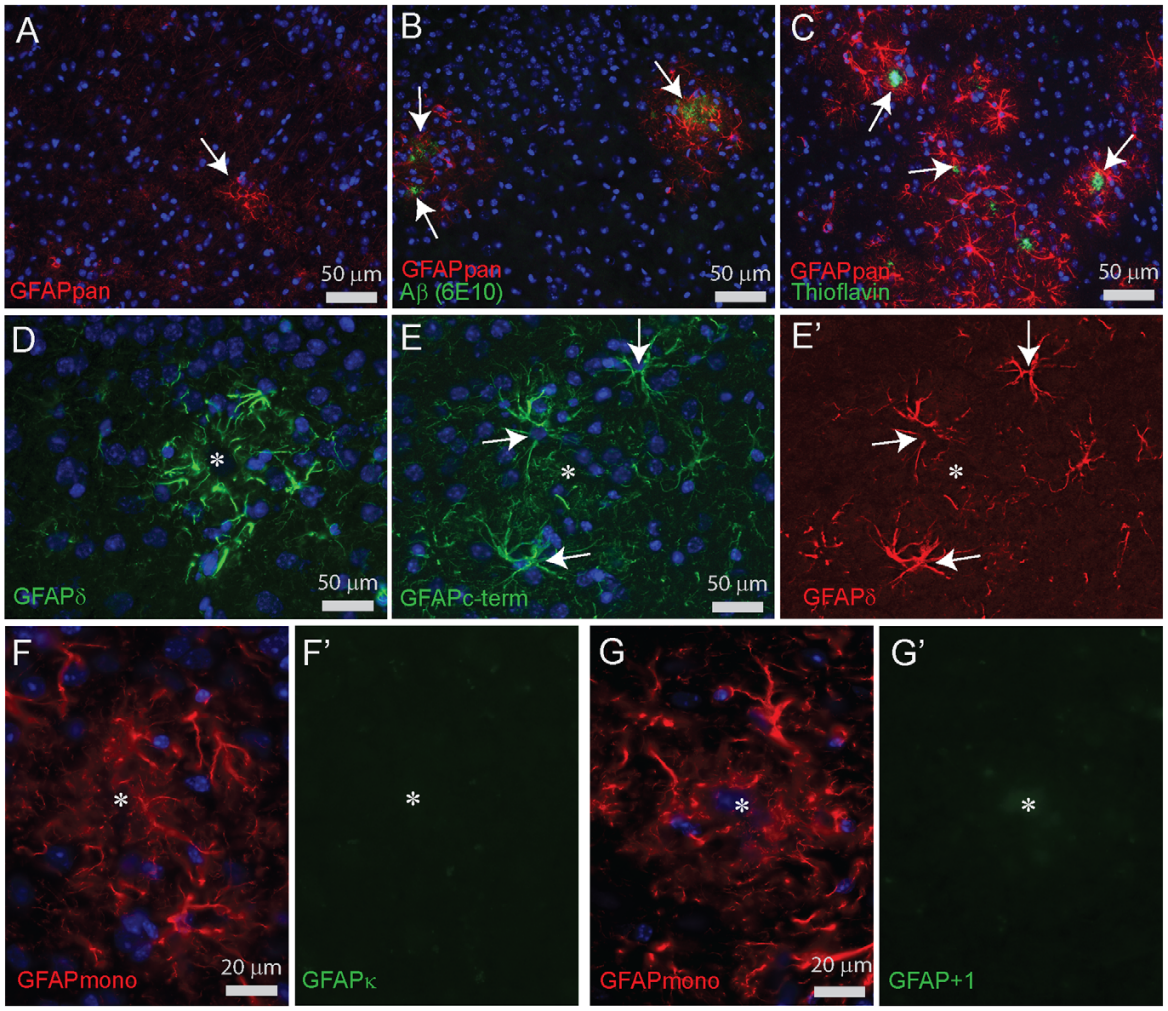
Immunocytochemical stainings for GFAP in cortex of APPswePS1dE9 mice. (**A**) GFAPpan staining in a WT mouse cortex showing a faintly stained network of fine filaments and a single astrocyte with an intense staining (arrow). (**B)** GFAPpan staining in the cortex of a 6 month APPswePS1dE9 mouse with amyloid deposits (arrows), detected by Aβ staining (6E10), illustrating the close spatial association of amyloid and increased GFAP staining. (**C**) GFAPpan staining in the cortex of a 9 month APPswePS1dE9 mouse showing that the reactive gliosis remains associated with plaques visualized by Thioflavin-S staining (arrows). (**D**) GFAPδ staining is strongly enhanced in reactive astrocytes around a plaque identified by a diffuse staining in the DAPI channel (asterisk). (**E,E’**) Double staining of the goat polyclonal GFAPc-term and rabbit polyclonal GFAPδ demonstrating the perfect overlap of GFAPα and GFAPδ localization in reactive astrocytes (arrows) around a plaque (asterisk) in a 9 month old APPswePS1dE9 mouse. (**F,F’**) Double staining of GFAPmono and GFAPκ showed no GFAPκ specific staining around a plaque in a 9 month old APPswePS1dE9 mouse. (**G,G’**) Double staining of GFAPmono and GFAP+1 showed no GFAP+1 specific staining around a plaque in a 9 month old APPswePS1dE9 mouse.

Immunostaining with the GFAPpan antibody revealed a network of fine weakly-stained filaments in the neuropil of the cortex of WT animals of all ages. Some individual astrocytes had a higher GFAP expression (Figure 11A). In 3 month old APPswePS1dE9 mice, neither plaques nor reactive astrocytes were observed. At 6 months and later, Thioflavin- and Aβ-positive deposits of various sizes were found and these were all associated with one or more GFAP-positive reactive astrocytes; while at further distance from plaques, a WT-like pattern was preserved (Figure 11B,C). In aged APPswePS1dE9 mice of 15 months and older, reactive astrocytes filled the complete cortex [38]. GFAPδ is hardly expressed in the WT cortex (Figure 9E), but is prominent in reactive astrocytes around all plaques at all ages (Figure 11D,E' and Figure S1). The staining pattern of GFAPδ in these reactive astrocytes of APPswePS1dE9 mice overlaps with that of the increased GFAPc-term staining (Figure 11E,E'). GFAPκ staining patterns could not be detected in WT or APPswePS1dE9 mice despite several permutations of fixation, epitope retrieval and staining procedures (Figure 11F,F'). The GFAP^+1^ antibody did not detect any immunopositive cells in either WT or APPswePS1dE9 brains (Figure 11G,G'). Plaque deposition in the hippocampus was associated with an increased staining intensity of GFAPpan, GFAPc-term and GFAPδ, while GFAP^+1^ and GFAPκ staining was not detected (data not shown).

**Figure 12:**
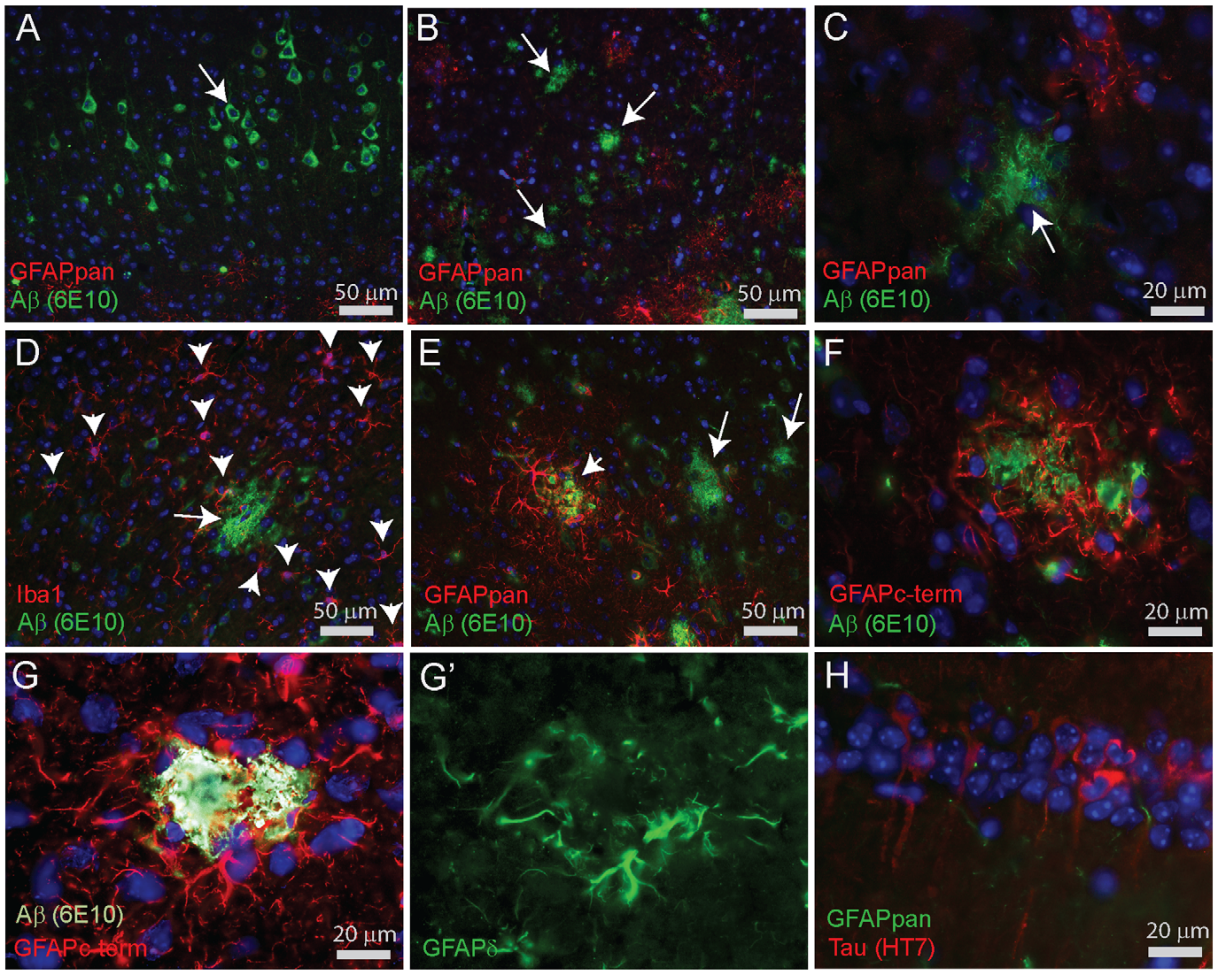
Immunocytochemical stainings for GFAP in cortex of 3xTgAD mice. (**A**) Intraneuronal APP/Aβ staining in neocortical neurons in layer 4/5 of 3xTgAD at 18 months. GFAPpan immunostaining does not show any reactive astrocytes. (**B**) Diffuse plaques (arrows) in the cortex of an 18 month old 3xTgAD female mouse are not surrounded by GFAPpan-positive reactive astrocytes. (**C**) Higher magnification of a cortical plaque (arrow) illustrating the absence of reactive gliosis. (**D**) Double staining for Aβ and microglia (Iba1) demonstrates the absence of microgliosis around a diffuse plaque (arrow). Arrowheads indicate the positions of individual Iba1-positive microglia without aggregation around plaques. (**E**) Some plaques in the cortex have a more compact amyloid structure (arrowhead) than the more diffuse plaques (arrows) and these deposits are surrounded by GFAPpan-positive reactive astrocytes. (**F**) Higher magnification of gliosis, demonstrated by GFAPc-term immunostaining around a compact plaque. (**G,G’**) Triple staining for Aβ and GFAPc-term and GFAPδ. Reactive astrocytes contacting plaques in 3xTgAD cortex are immunopositive for GFAPc-term and GFAPδ. (**H**) Hippocampal neurons with accumulated tau-protein (HT7 antibody) in 3xTgAD are not associated with reactive astrocytes.

In the cortex of 3xTgAD mice of 3 months and older, intraneuronal APP/Aβ staining in some neurons in layer 4/5 was noticed. These neurons were never surrounded by reactive astrocytes (Figure 12A). 3xTgAD mice of our colony display cortical plaques only at the age of 18–21 months and older. These are predominantly diffuse plaques composed of a dense network of fine amyloid fibrils and were neither associated with reactive astrocytes (Figure 12B,C,E) nor with activated microglia (Figure 12D). However, the less frequently observed dense-cored compact plaques were surrounded by GFAPpan-positive reactive astrocytes (Figure 12E,F,G). That said, compared to APPswePS1dE9, gliosis in the 3xTgAD model is less vigorous. Reactive astrocytes around dense-cored plaques were GFAPc-term- and GFAPδ-positive (Figure 12G,G'). In 3xTgAD mice, neither GFAPκ nor GFAP^+1^ staining were detected (data not shown). All our findings were confirmed in sections of 3xTgAD mice obtained from the other colony which showed earlier plaque formation at around 12 months of age. In the hippocampus, tau-positive pyramidal neurons were not associated with gliosis (Figure 12H).

### Vimentin-, Nestin-, and Synemin-immunostaining in cortex of WT and Around Plaques in AD Mice

**Figure 13:**
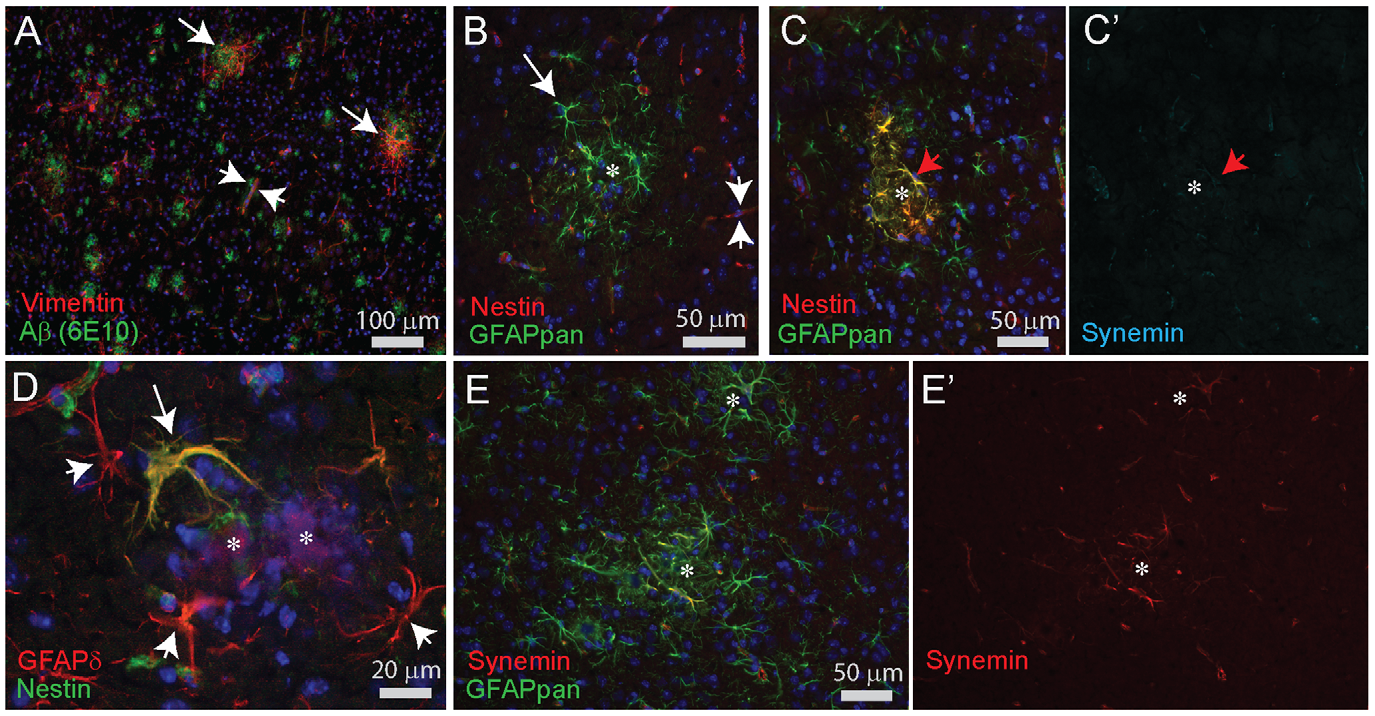
Immunocytochemical stainings for Vimentin, Nestin, and Synemin in cortex of APPswePS1dE9 mice. (**A**) Vimentin (VIM) immunostaining does not associate with all plaques detected by Aß (6E10) staining (15 month old animal). Only some of the plaques are surrounded by Vimentin-positive astrocytes (long arrows). Most Vimentin staining is located around blood vessels (short arrows). (**B**) Double staining of GFAPpan and nestin in a 6 month old mouse shows the absence of Nestin in most of the reactive astrocytes around plaques (asterisk). Nestin staining is mostly found associated with blood vessels (short arrows). (**C,C’**) A triple staining for Vimentin, GFAPpan and Synemin illustrating a rare example of enhanced nestin expression in GFAPpan-positive astrocytes around a plaque (asterisk) while Synemin expression (**C'**) is absent (9 months). (**D**) Double staining for GFAPδ and nestin reveals that not all GFAPδ-positive reactive astrocytes around a plaque (asterisk) are nestin positive (short arrows) but some are (arrow). (**E**) Reactive astrocytes near plaques (asterisks) display only enhanced levels of GFAPpan that are only occasionally weakly positive for Synemin in a 9 month old mouse. Most Synemin staining is located around blood vessels. Ependymal cells were always strongly positive for Synemin in the same sections (not shown).

In WT mice, vimentin staining was localized mostly in blood vessels. In APPswePS1dE9 mice, vimentin was also expressed by hypertrophic astrocytes around larger-sized plaques. These vimentin positive cells only became apparent at 9 months and were more numerous thereafter (Figure 13A). Nestin was mainly expressed in blood vessels in the parenchyma. Most reactive astrocytes did not express nestin (Figure 13B), but a few large-sized nestin-positive astrocytes were noticed with increased GFAPδ levels (Figure 13D). Rarely, all reactive astrocytes around a plaque were nestin-positive, but these cells were never synemin-positive (Figure 13C,C'). In WT, synemin immunostaining was localized in blood vessels and in APPswePS1dE9 mice only the larger hypertrophic astrocytes around plaque displayed a very faint immunolabeling (Figure 13D).

## Discussion

The aim of the study described here was to characterize the expression pattern of different GFAP isoforms in WT mice and in the context of AD-related gliosis in transgenic mice. To this end we developed isoform-specific qPCR assays, expanded our existing collection of antibodies against human GFAPδ and GFAP^+1^ [24], [25] with their mouse equivalents, and developed new antibodies against msGFAPκ [26]. Transcripts for Gfap-α, -β, -γ, -δ, -κ, and Gfap-ζ were detected with a steady expression profile in different mouse brain areas, with Gfap-α, -δ, -ζ, and Gfap-κ being most abundant. This pattern did not change in the cortex of adult mice between 3 and 18 months. Plaque-deposition evoked reactive gliosis in two AD-mouse models. This reactive gliosis was accompanied by significant increases of *all* Gfap isoforms, thereby leaving the stoichiometry of the isoforms essentially intact. At the protein level, our panel of GFAP antibodies detected GFAPα and GFAPδ at overlapping patterns in all brain areas, including the SVZ; while GFAP^+1^ and GFAPκ could not be found in mouse brain. Like in the human brain, high GFAPδ expression was observed in the SVZ and RMS. We also localized GFAPδ in neurogenic radial astrocytes of the SGZ. However, mouse GFAPδ is not restricted to these areas and is also expressed at low levels throughout the brain e.g. by hippocampal astrocytes and near the pial surface. In reactive astrocytes around plaques, a parallel increase of GFAPα and GFAPδ was observed. In contrast to the human brain, frameshifted GFAP^+1^ isoforms do not mark a specific astrocyte subtype [8], [24], [28], [29].

### Characterization of GFAP Isoforms in Mouse

At the transcript level in mouse brain, the expression of Gfap-α (set at 100%) is by far the most abundant followed by Gfap-δ (7.9%), Gfap-ζ (4.5%), Gfap-κ (1.0%), Gfap-γ (0.3%), and Gfap-β (0.008%). We did not observe any age-dependent changes in the cortex between 3 and 18 month for either transcript or protein levels. The isoform termed by us as Gfap-ζ (zeta) was already noted in mouse tissue as documented by Zelenika and colleagues (1995). We show now, for the first time, that Gfap-ζ mRNA is readily detectable in mouse brain. Further research into this isoform awaits the identification of the full-length transcript. Gfap-ΔEx7 is a hitherto undescribed isoform tentatively encoding for a GFAP^+1^ isoform in mouse brain. However, the functional relevance of Gfap-ΔEx7 remains to be established as transcript levels are extremely low. Transcript levels of isoforms previously identified in human AD tissue and epileptogenic lesions (GFAPΔ135, GFAPΔ164, GFAPΔEx6) were not detected in either WT or AD mice [24], [28], [29]. In line, GFAP^+1^ staining was undetectable and western blots showed no GFAP^+1^ protein.

From our panel of isoform specific GFAP antibodies, only GFAPc-term and GFAPδ displayed staining in mouse sections and blots, we therefore conclude that only GFAPα and GFAPδ isoforms are expressed at the protein level in mouse brain. However, it cannot be excluded that GFAPβ, GFAPγ or GFAPζ may still contribute to the GFAPpan immunostaining, however more sequence information of these isoforms is needed so that specific antibodies can be developed and more definite conclusions can be drawn.

Concerning Gfap-κ, the qPCR assay on mouse brain cDNA clearly demonstrated the presence of this transcript. A PCR fragment from the start codon in exon 1 up to the Gfap-κ specific 390 bp region in intron 7–8 was isolated from mouse brain cDNA with a nucleotide sequence conform the GenBank entry. It should be noted that a previously published sequence on Gfap-κ contains several mismatches with the GenBank deposit, resulting in a different reading frame of the C-terminal domain [26]. All antibodies raised against the deduced GFAPκ C-terminus yielded a positive immunostaining on Gfap-κ transfected cells as well as on western blots of these cells. At present, we can only speculate why the GFAPκ antibodies do not show a staining pattern on mouse brain sections or on westerns blots. It seems logical to conclude that Gfap-κ transcripts are either not translated or that the protein is short-lived in astrocytes *in vivo*, being rapidly degraded after synthesis by cellular quality control mechanisms [44]–[46].

Previous work has shown that the unique tail domains of the GFAP isoforms lead to differences in the ability to form stable filamentous networks [25], [26], [37], [47]. Our transfection studies confirm that, comparable to hsGFAPα, also msGFAPα has the best intrinsic capacity to form filaments, while all other isoforms yield compromised networks. The estimated transcript ratio of msGfap-δ to -α *in vivo* is about 1∶ 13, but whether the resulting level of GFAPδ expression has any effects on the IF network morphology in astrocytes, as shown for co-transfection of hsGfap-α and -δ, is subject of ongoing research [25], [37], [47].

### GFAPδ Isoform Expression is Increased in the SVZ and RMS

In the human brain, GFAPδ is preferentially expressed by astrocytes in the SVZ, RMS, and olfactory bulb [25], [31]. Moreover, astroglial cells can also transform into tumor cells forming astrocytomas, which are the most common type of tumors in the human brain; with high grade astrocytomas expressing higher levels of GFAPδ [33], [34], [48]. Subsequent work from our group has shown that GFAPδ-positive cells display astrocyte-specific markers, the neural stem cell marker nestin, and several proliferation markers. Such a profile is compelling evidence for an expression in neurogenic astrocytes [31]. The msGFAPδ antiserum revealed a differential expression throughout the mouse brain with low to undetectable levels in cerebellum and neocortex but clear expression in astrocytes lining the pial surface, in SVZ and RMS, always together with a strong GFAPα expression. Such a differential GFAPδ expression was not predicted by tissue transcript levels. A notable difference with the GFAPδ localization in human SVZ is that the GFAPδ staining in humans is more perinuclear which enables identification of GFAPδ co-localization with nuclear proliferation markers [31]. Consequently, we could not unambiguously associate GFAPδ in proliferating cells marked by BrdU incorporation or Ki67. Nevertheless, the observed double staining of GFAPδ with nestin is consistent with the view that msGFAPδ is, as hsGFAPδ, expressed in neural stem cells. The co-expression of GFAPδ next to GFAPα in these cells could potentially lead to changes in cell division, migration, and presenillin-associated pathways [31], [47], [49], [50].

### GFAP-isoform Expression in AD-mouse Models

Amyloid plaques in human AD and in AD mouse models are surrounded by reactive astrocytes with an increased GFAP expression [8], [38], [51]. GFAP levels also correlate inversely with cognitive function [52], [53]. Astrocytes are involved in a wide range of functions primarily aimed at maintaining brain homeostasis. The proximity of plaques may lead to abnormalities in some of the astrocyte-mediated functions, [54] such as the degradation of Aβ deposits [55], [56], calcium signaling [57], [58], metabolism [59], [60], gap junctional communication [61], synaptic functions [62], and glutamate uptake [6]. An increase of GFAP levels in astrocytes is a generally accepted marker for astrogliosis and is highly associated to plaque load, and to a lesser extent to the number of neurofibrillary tangles [63]–[66]. The reaction of astrocytes is dependent on the presence of GFAP, since GFAP^−/−^ astrocytes do not react by changing morphology and fail to form a barrier-like structure around Aβ deposits in hippocampus slice cultures [20]. The precise relation to plaque-associated reactive gliosis and the reported functional changes in astrocytes is not well understood. On the one hand, the transformation to reactive astrocytes may have detrimental effects by increasing neurotoxic substances, exacerbating cell loss, and minimizing CNS repair by scar formation. But on the other hand, reactive gliosis has also been regarded as beneficial by enclosing the affected area, restricting inflammation, taking up excessive amounts of extracellular glutamate, and eliminating free radicals [1], [60], [67]–[69].

In APPswePS1dE9 mice at an age over 6 months, severe gliosis occurs around each plaque [38] and this is accompanied by a significant increase of transcript levels of all detectable Gfap-isoforms with no differential change in the expression profile of the various isoforms. In the 3xTgAD model, the increase in Gfap isoforms was less pronounced, but again no differential changes were noted. Immunostainings demonstrated a very strict association of plaques and GFAP upregulation in the cortex of APPswePS1dE9 mice. Already diminutive amyloid deposits present at 6 months are linked to a GFAPα and GFAPδ increase in reactive astrocytes. In the temporal cortex of human AD donors, transcript levels for Gfap-α were significantly increased 2.4 fold and for Gfap-δ a lesser 1.5 fold. Immunostainings revealed only a minor increase of GFAPδ staining [25]. In AD mice, we did not observe such a differential increase.

In sharp contrast to the astrogliosis in APPswePS1dE9 model, most plaques in the 3xTgAD model have a structure composed of rather loosely organized fibrils and these deposits do not trigger reactive astrogliosis or an activation of microglia. In an early characterization of the 3xTgAD model, GFAP levels were assessed by western blots and found to be increased by approximately 75% [40]. More in line with our findings, Mastrangelo and colleagues reported a weak microglia response and the absence of clear changes in GFAP staining in 3xTgAD mice [70]. However, the other much less frequently observed type of plaque in the cortex of 3xTgAD mice has a more compact core with an amorphous structure. This denser type of plaque was associated with reactive astrocytes. Plaque formation in the 3xTgAD mice starts at a much later age (around 21 months) suggesting a lower degree of Aβ overproduction compared to the APPswePS1dE9 which may lead to a different pattern of amyloid aggregation and, in turn, may not to trigger a response by astrocytes and microglia. It is of interest to note that the differential response to the different types of deposits is very much similar to the AD-related gliosis found in humans where diffuse plaques do not trigger gliosis while dense-cored plaques are associated with reactive astrocytes [6]. It has been reported that in 3xTgAD mice astrocytes near plaques had the typical reactive phenotype whereas astrocytes further away showed signs of glial atrophy evidenced by a decreased GFAP volume and a reduced arborization [51], [71]. Because we focused our study on the cortex and as GFAP levels are typically low in most cortical astrocytes, we are unable to draw any conclusions on such changes.

An interesting finding is the observation is that reactive gliosis in APPswePS1dE9 mice is not associated with a concerted increase of GFAP, nestin, vimentin, and synemin as has been found to occur after different forms of experimental brain injuries [72]–[74]. Vimentin was found to increase only following GFAP induction and only in the more hypertrophic reactive astrocytes, nestin was hardly ever found increased, and we never observed an upregulation of synemin. The qPCR assays were in good agreement with this pattern of differentially regulated expression. In mouse AD models, IF upregulation has not been well documented. In human AD tissue, vimentin is strongly increased in hypertrophied astrocytes near plaques but not all plaques appear to be associated with vimentin staining and the number of vimentin-positive astrocytes was estimated to be less than 10% of GFAP-positive astrocytes [75], [76]. The IF nestin is highly expressed in multipotential stem cells of the developing brain and is down-regulated in the adult brain, except in neurogenic niches. Reactive gliosis as a result of different experimental brain injuries induces the re-expression of nestin [72], but we here report that this does not happen in the context of amyloid-induced gliosis in the APPswePS1dE9 mice. Possibly the degree of gliosis may not be severe enough to trigger nestin expression which is in line with our observations that astrocytes do not become proliferative in this model [1], [38], [72]. In summary, reactive astrocytes seem to upregulate both GFAPα and -δ levels to a similar extent while the increase in expression of the other IFs is much more heterogeneous. Whether this has implications, for instance on astrocyte motility, requires further study [77].

In conclusion, the here presented study shows that the expression of the GFAPδ isoform in mouse SVZ is homologous to the situation in human SVZ and RMS and offers a more accessible model to study the functional role of GFAPδ in neurogenesis in the adult brain *in vivo*. Mouse GFAPδ is also involved in reactive gliosis and may be implicated in the morphological alterations of reactive astrocytes are going through. In contrast, the expression of GFAP^+1^ in a subclass of astrocytes in human brain is not reproduced in WT or AD mouse brain and requires further studies on the human brain to unravel their function and possible involvement in neuropathological conditions.

## Materials and Methods

### Mouse Brain Material

#### Isolation of brain regions for GFAP-isoform transcript level assessment and western blots

For the isolation of RNA from different brain areas, 8 week old male C57BL/6 mice (n = 7) were sedated by CO_2_/O_2_ and decapitated. The brains were frozen over dry ice, 50 µm thick coronal cryosections were cut, mounted on poly ethelene napthalate (PEN) foil covered slides (Carl Zeiss Ltd., United Kingdom), and after drying stored at −80°C. No additional fixation step was used, as fixation steps have been shown to reduce the mRNA yield significantly.

Specific brain areas were isolated using laser dissection microscopy (LDM) using a PALM/Zeiss Microbeam system. For each area, 4–7 sections were used and the regions of interest were directly collected in tubes containing 1 ml of TRIzol (Life Technologies). Regions of interest were: (i) Cerebellum; a circle with a diameter of 1250 µm was isolated from the cerebellar cortex. (ii) Neocortex; a circle with a diameter of 1250 µm was isolated from the same coronal sections as used for SVZ isolations. (iii) SVZ; a 380 µm wide zone of tissue adjacent to the lateral ventricle. (iv) SVZ-Striatum; the area 140 µm lateral to the area selected as SVZ. (v) Striatum; the area 140 µm lateral to the SVZ-Striatum. (vi) Dentate gyrus; an area delineated by 100 µm around the SGZ. (vii) CA1; a circle with a diameter of 900 µm encompassing the CA1 area of the hippocampus.

For macrodissection of the subventricular zone (SVZ), 13–15 month old mice (n = 9) were sedated with pentobarbital and decapitated. Two coronal slices including the lateral ventricles were hand-cut. The lateral wall of the lateral ventricle was dissected as a thin strip of tissue, next to samples from striatum, and cortex. Samples were used for RNA isolation to determine transcript levels and to prepare protein homogenates for western blotting.

To label proliferating cells some mice were injected with 150 mg/kg 5′-bromo-2′-deoxyuridine (BrdU) and were sacrificed either 2 h or 4 weeks later [38].

### Transgenic Mouse Models for AD

APPswePS1dE9 double-transgenic mice (AD) were studied (Jankowsky et al., 2004). For details see The Jackson Laboratory [strain B6C3-Tg(APPswe,PSEN1dE9)85Dbo/J; stock number 004462; http://jaxmice.jax.org/]. The mice were kindly provided by Dr. D. Borchelt. These mice express a chimeric mouse/human APP containing the K595N/M596L Swedish mutation and a human PS1 variant carrying the exon 9 deletion both driven by mouse prion promotor elements, directing the expression to neurons (Jankowsky et al., 2004). Heterozygous mice were maintained by crossings with wild-type (WT) C57BL/6 mice. WT littermates served as age-matches controls. The first Aβ-plaques are found between 4 and 5 months of age and the number of plaques in the cortex increases gradually over time with no clear difference between males and females [38].

Transgenic mice and control WT littermates were sacrificed at different ages: 3, 6, 9, 12, 15, 18 months. For RNA isolation, animals were sedated by CO_2_/O_2_ and decapitated. The cortex was dissected, frozen in liquid nitrogen and stored at −80°C. For each age group at least 6 WT and 6 transgenic mice were studied. For immunofluorescence, mice were fixed by transcardial perfusion with 4% paraformaldehyde in phosphate buffered saline (PBS, pH 7.4), the brain was isolated, post-fixed for two hours and rinsed in PBS. The brains were placed in 20% sucrose-PBS overnight and frozen over dry ice. Alternatively, the brain was isolated from non-perfused mice and directly frozen over dry ice. For each fixation protocol at least 4 WT and 4 transgenic mice were used.

Homozygous 3xTgAD female mice were used for immunofluorescence to study gliosis. The 3xTgAD line, kindly provided by Dr. F. LaFerla, was originally generated by co-microinjection of human APP (K670M/N671L) and tau (P301L) transgenes under the control of the Thy 1.2 promoter into mutant PS-1 (M146V) knock-in mice [38], [78]. 3xTgAD and control mice with the same genetic background (3xTgWT) were studied at the age of 21 and 24 months. In our hands, this line develops cortical plaques only after 21 months which is at later age than described originally [78] and more in line with the development reported by others [70]. For comparison, we also studied material from a colony of 3xTgAD mice developing plaques at an earlier age of 12 months (J.J.R.). These mice were perfused with 25 ml 3.75% acrolein (TAAB, UK) and 2% paraformaldehyde in 0.1 M phosphate buffer (PB) pH 7.4, followed by 75 ml 2% paraformaldehyde [71]. Coronal sections of the brain were cut into 40–50 µm thick vibratome sections. Free-floating sections were processed for immunofluorescence following the same protocol as described for cryosections mounted on slides.

All animals were housed under standard conditions with access to water and food ad libitum. Animal handling and experimental procedures were reviewed and approved by the ethical committee for animal care and use of experimental animals of the Royal Netherlands Academy for Arts and Sciences, acting in accordance with the European Community Council directive of 24 November 1986 (86/609/EEC). All efforts were made to minimize the degree of discomfort and number of animals used for the study presented here.

### Laser Microdissection and Pressure Catapulting (LMPC) of Plaques

Samples were isolated from 14 µm cryosections of directly frozen brains of 6 and 9 month old WT and APPswePS1dE9 mice. Sections were mounted on PEN foil covered slides as described above. Slide boxes were equilibrated to room temperature. Slides were washed for 10 min in 70% EthOH, incubated for 10 min in 0.0125% Thioflavin-S in 70% EthOH. Thioflavin-S is a fluorescent derivative that exhibits high affinity for Aβ- aggregates. Slides were washed for 5 min in 70% EthOH, dried and subjected to LMPC using a PALM/Zeiss Microbeam system. The catapulted material was collected in AdhesiveCaps (Zeiss). Forty plaques and forty non-plaque regions were isolated from the cerebral cortex of transgenic animals (6 month, n = 5; 9 month, n = 7), as well as forty plaque-sized regions from WT littermates (6 month, n = 5; 9 month, n = 8). The area excised consisted of the Thioflavin-S positive area together with a border of surrounding tissue known to contain the nuclei of reactive astrocytes [38]. The catapulted area had a total diameter twice that of the Thioflavin-S area. The number of successfully catapulted areas was verified after isolations by visualization of the adhesive cap plane and counting the number of adhered samples. RNA was isolated using TRIsure (Bioline) and an overnight (O/N) precipitation in isopropanolol supplemented with 20 µg glycogen (Life Technologies). No effort was made to determine the RNA yield from these samples and the total yield was immediately used for cDNA synthesis followed by qPCR assays as described above. Differences in starting amount of RNA were corrected used Gapdh, Hprt, Actb, Ef1a2 transcript levels as reference.

### RNA Isolation-cDNA Synthesis - qPCR

From mouse brain, RNA was isolated from the cortex of one hemisphere. RNA from mouse cortex and microdissected brain areas was isolated using TRIzol (Invitrogen) or TRIsure (Bioline) and an O/N precipitation in isopropanolol. The quality of the RNA was determined on a Bioanalyzer 2100 (Agilent Technologies). Total RNA (1.0 µg for cortex, or the total yield of LDM or LMPC samples) was DNase treated and used as a template to generate cDNA following the manufacturer’s instructions (Quantitect-Qiagen) with a mix of oligo dT and random primers. Incubation was for 30 min at 42°C. The resulting cDNA was diluted 1∶20 and served as a template in real-time quantitative PCR assays (SYBR® Green PCR Master Mix (ABI). See for technical details on quantification and normalization procedures [38], [79].

### Primers

Sequences of primers used and information on their design are given in Table S1.

### Immunofluorescence

Cryosections (10 µm) were mounted on Superfrost Plus slides (Thermo Scientific), fixed for 10 min with 4% paraformaldehyde in PBS, washed, and blocked with 10% normal donkey serum +0.04% Triton X-100 in 0.05 M phosphate buffer for 1 hour. Incubation with the different GFAP-isoform specific antibodies was carried out O/N at RT in 3% normal donkey serum +0.04% Triton X-100 in 0.05 M phosphate buffer. Immunostaining was visualized with 1∶1400 diluted Cy3- or DyLight488-conjugated secondary antibodies (Jackson ImmunoResearch Laboratories) incubated for 2 hours in 0.05 M phosphate buffer at room temperature. Sections were washed and coverslipped in Vectashield with DAPI (4',6-diamidino-2-phenylindole) added as a nuclear dye (Vector Laboratories). Plaques were localized with Thioflavin-S or by antibodies against Aβ (6E10-Signet/Covance).

Cultured U343 or SW13/cl.2 cells were cultured on gelatin-coated coverslips, rinsed with PBS and fixed for 15 min with 4% paraformaldehyde in PBS. Immunostaining was carried out as described above for cryosections.

### Cloning Mouse GFAP Splice Variants

Full-length Gfap-α and Gfap-δ were isolated by PCR from C57Bl/6 mouse brain cDNA (for primer sequences see Table S1). PCRs to obtain full length GFAPκ (#1065-#1067) generated always the 390 bp shorter GFAP-δ product. For Gfap-κ, a 1346 bp PCR fragment was obtained from mouse cortex cDNA using primers targeting exon 1 (#1065) and intron 7/8 (#1088) at position 150–170 located in the 390 region specific for Gfap-κ. The fragment was digested with NotI and EcoRI and ligated into pcDNA3. The sequence of the 390 bp Gfap-κ specific sequence matched completely with the deposits in GenBank and Ensembl and predicts a unique 46 aa C-terminal domain before hitting a stop-codon. It should be noted that the published sequence by Blechingberg et al. represents the sequence known under Mm_Celera 232000009822206 that has several mismatches with the GenBank deposit, resulting in a different reading frame of the C-terminal domain [26]. Expression plasmids containing full length Gfap-ΔEx6, Gfap-Δ164, Gfap-Δ135, and Gfap-ΔEx7 were constructed by modifying the pcDNA3-Gfap-α full-length plasmid.

### Cell Culture and Transfections

The human adrenal carcinoma cell line SW13/cl.2 [80] was maintained in DMEM/F-12 medium (Invitrogen), supplemented with 10% heat-inactivated fetal calf serum (Invitrogen) and penicillin/streptomycine, and maintained in a humidified environment at 37°C with 5% CO2. The human astrocytoma cell line U343 [81] was maintained in DMEM high glucose/HAM F-10 (1∶1) medium (Invitrogen), with 10% heat-inactivated fetal calf serum and penicillin/streptomycine. SW13/cl.2 cells do not express IFs while U343 cells express vimentin, nestin and GFAP [14].

For transfection and immunostaining purposes, cells were cultured in 24 well plates on coverslips coated with 0.2% gelatin. The next day, medium was refreshed one hour prior to transfection using polyethylenimine (PEI). For single transfections, 0.4 µg DNA/25 µl medium without antibiotics was used per well. For double transfections, SW13/cl.2 cells were transiently transfected with pIRESmsGFAPα-EGFP and pIRESmsGFAPδ-mCherry using PEI (0.4 µg DNA and 1.6 ul PEI 1 mg/ml). Cells were fixed in 4% PFA for 15 min 24 h after transfection.

### Antibodies

Antibodies used in this study are listed in Table 3. Based on the differential sequences in the C-terminal domains of the GFAP isoforms, new antibodies against specific sequences in mouse msGFAPδ (Gfap transcript variant 1; NM_001131020.1), msGFAPκ, and msGFAP^+1^ were raised in rabbits. BLAST searches did not show significant homologies with other proteins. The peptide was coupled to thyroglobulin or Keyhole Limpet Hemocyanin in a 1∶1 ratio using glutaraldehyde, dialyzed against 2% sodium metabisulphite pH 6.5. After collecting pre-immune serum, rabbits were injected with 2–5 mg of coupled peptide mixed with either Specol or Freund’s Complete Adjuvant followed by 2–3 booster immunisations. Some of the antisera were immunopurified using the peptide coupled to Sepharose 4B.

### Western Blots

To obtain recombinant protein samples for western blotting, pcDNA3 expression plasmids for Gfap-α, Gfap-δ, Gfap-κ, Gfap-Δ135, Gfap-Δ164, Gfap-ΔEx6, and Gfap-ΔEx7 were transfected separately into SW-13 cl.2 cells. Protein was isolated from these cells by homogenization with lysis buffer (0.1 M NaCl, 0.01 M Tris-HCl pH 7.6, 1 mM EDTA pH 8.0) supplemented with a protease inhibitor cocktail (Roche). The samples were dissolved in 2× loading buffer (2×: 100 mM Tris, 4% SDS, 20% glycerol, 200 mM DTT, 0.006% bromophenol blue) and boiled for 5 minutes. Subsequently, the samples were run on a 7.5% SDS-PAGE gel and blotted to nitrocellulose paper. Blots were probed overnight with primary antibodies (see Table 3 for details) diluted in Supermix (0.05 M Tris, 0.9% NaCl, 0.25% gelatin, and 0.5% TritonX-100, pH 7.4). The next day, the blots were washed with Tris-buffered saline with Tween (TBS-T; 100 mM Tris-HCl pH 7.4, 150 mM NaCl, with 0.2% Tween-20), incubated for one hour at room temperature with secondary antibodies IRDye800-conjugated (1∶5,000; Rockland Immunochemicals Inc., Gilbertsville, USA) and, for actin probing, Cy5-conjugated donkey-anti-mouse (1∶2,000; Jackson ImmunoResearch Laboratories) diluted in Supermix. After three washes in TBS-T, bands were visualized with the Odyssey Infrared Imaging System (LI-COR Biosciences, Lincoln, USA).

Protein samples were homogenized from frozen macrodissected tissue samples or from isolated cortex of transgenic and WT mice brains, using an Ultra Turrax. Samples were prepared in 50 mM Hepes, 250 mM sucrose, 5 mM MgCl2, 0.5 mM DTT, 40 mM KCl, pH 7.4 followed by sequential centrifugation steps transferring the supernatant over to each next step (4°C for 10 min at 1300 g, 10 min at 10,000 g, and 25 min at 13,000 g). Pellets of the last two centrifugation steps were collected in 20 mM Tris-HCl, 1% Triton-X100, pH 7.4. The pellets were dissolved by shaking for 1 h at 4°C and pooled. The protein concentration of the supernatant and pellet sample was determined with the BCA protein assay kit according to the manufacturers protocol (Thermo Fisher Scientific) in a Varioskan Flash reader (Thermo Fisher Scientific). Samples were dissolved in 2x loading buffer, boiled for 5 minutes, loaded on a 7.5% SDS-PAGE gel and blotted as described above.

### Statistical Analyses

Data was analyzed for significance using GraphPad Prism software (GraphPad Software, San Diego, CA, USA). Distributions were tested for normality. When normality was assumed, the parametric unpaired Student's t-test was used for to test for significant differences between groups. When normality was not assumed, the non-parametric Mann Whitney U-test was used. Differences were considered to be significant at P<0.05.

## Supporting Information

Figure S1 **Immunocytochemical stainings in SVZ and cortex of an APPswePS1dE9 mouse.** (**A,B**) Double staining of GFAPc-term and GFAPδ in SVZ and (**C,D**) cortex of a 9 month old APPswePS1dE9 mouse. Photomicrographs in A,B were obtained from the same section and recorded with identical settings as in C,D. In the SVZ, GFAPc-term and GFAPδ show an identical distribution but GFAPδ staining is more intense. In the cortex reactive astrocytes around a plaque (asterisk) display high-intensity staining for both GFAPc-term and GFAPδ (arrows).
(TIF)

Table S1 **Sequences and characteristics of primers of GFAP splice-variants, other intermediate filaments and reference genes.**
(DOCX)
