# Supplementary figures and images for: GFAP Isoforms in Adult Mouse Brain with a Focus on Neurogenic Astrocytes and Reactive Astrogliosis in Mouse Models of Alzheimer Disease
Source: PLoS One. 2012 Aug 13;7(8):e42823. doi: 10.1371/journal.pone.0042823 (PMC3418292; doi:10.1371/journal.pone.0042823)

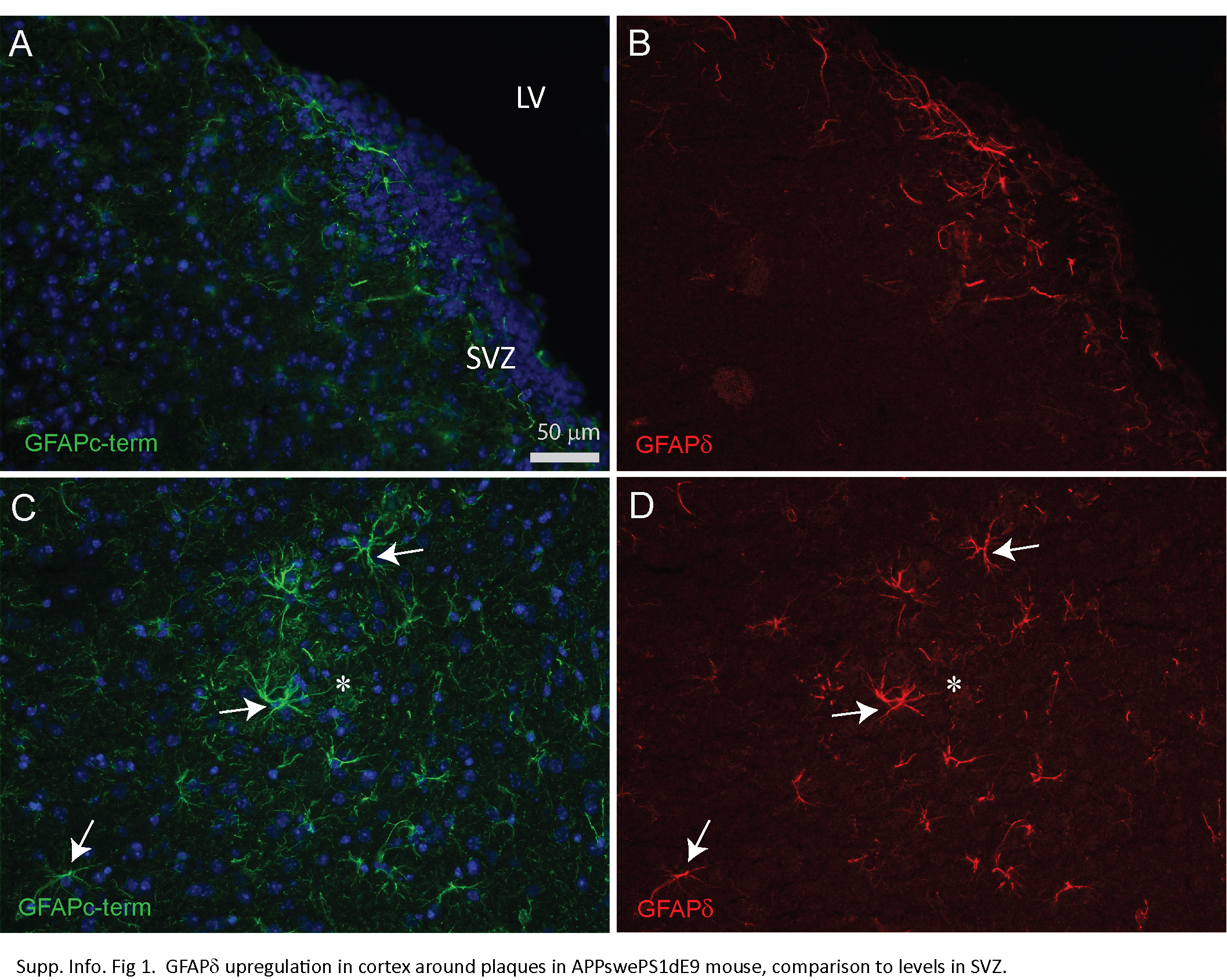

Supplement: Figure S1 — Immunocytochemical stainings in SVZ and cortex of an APPswePS1dE9 mouse. (A,B) Double staining of GFAPc-term and GFAPδ in SVZ and (C,D) cortex of a 9 month old APPswePS1dE9 mouse. Photomicrographs in A,B were obtained from the same section and recorded with identical settings as in C,D. In the SVZ, GFAPc-term and GFAPδ show an identical distribution but GFAPδ staining is more intense. In the cortex reactive astrocytes around a plaque (asterisk) display high-intensity staining for both GFAPc-term and GFAPδ (arrows). (TIF) [file pone.0042823.s001.tif]
