# Supplementary material for: GFAP Isoforms in Adult Mouse Brain with a Focus on Neurogenic Astrocytes and Reactive Astrogliosis in Mouse Models of Alzheimer Disease
Source: PLoS One. 2012 Aug 13;7(8):e42823. doi: 10.1371/journal.pone.0042823 (PMC3418292; doi:10.1371/journal.pone.0042823)
Supplement: Table S1 — Sequences and characteristics of primers of GFAP splice-variants, other intermediate filaments and reference genes. (DOCX) [file pone.0042823.s002.docx]

**Table** **S1.** Sequences and characteristics of primers of GFAP splice-variants, other intermediate filaments and reference genes.

| Gene | Forward | Reverse | Position | Amplicon  (bp) |
| --- | --- | --- | --- | --- |
| #1065 | ATGAATTCAGAGGCAGGGCAGGATGG |  | Exon1 |  |
| #1088 |  | GGAGCGGCCGCAGATGCATGCCCTAGGATCCT | Intr7/8 |  |
| WQ465 | AGCCTTCGTGACCCTCCAC |  | Intr7/8 |  |
| #1067 |  | CTGCGGCCGCGAGCCAGGACCTAGGGTG | Intr7/8 |  |
| Gfap-α | GGAGATGCGGGATGGTGAG | ACCACGTCCTTGTGCTCCTG | Ex8 - Ex9 | 57 |
| Gfap-β | CAGATTTAGTCCAACCCGTTCC | GCGTCTCCGCTCCATCC | UTR5’- Ex1 | 83 |
| Gfap-γ | CTTCTGCCACTTTACCGGTCC | TCTTGGGTCCCTGTCCTAACC | Intr1 | 78 |
| Gfap-δ | TCTCCAACCTCCAGATCCGA | TGACTTTTTGGCCTTCCCCT | Ex7 - Ex7+ | 60 |
| Gfap-κ | AGCCTTCGTGACCCTCCAC | AGATGCATGCCCTAGGATCCT | Ex7 - Ex7+ | 60 |
| Gfap-ζ | AGCAAACTGGGTCTGGGATTATT | TGCTCCTGCTTCGAGTCCTTA | Int8/9 – Ex9 | 92 |
| Gfap-Δ135* | CCCTGCGCGGCACGGAGT | GATGTCCAGGGCTAGCTTAACG | Ex5/6 – Ex6 | 54 |
| Gfap-Δ164 | GCACGAAGCTAACGACTATCGC | AGAAAGTCTGTACAGGAGTAACT | Ex5 – Ex6/7 | 145 |
| Gfap- ΔEx6* | GTCCCTGCGCGGCACGCATC | TTTGGTGTCCAGGCTGGTT | Ex6/7 – Ex8 | 78 |
| Gfap- ΔEx7 | CGAAGAAAACCGAACCAG | TGCTCCTGCTTCGAGTCCTTA | Ex6/8 – Ex9 | 123 |
| Synm-H | GGTCCTTTCGGCACATTCAA | GATACGGGTCTCCCGGTGA |  | 76 |
| Synm-M | AGAACAGGTGACCTTCGGAGG | ACTTCCGACCCTGTGTCTCG |  | 102 |
| Vim | TCCAGAGAGAGGAAGCCGAA | GCAAGGATTCCACTTTCCGTT |  | 102 |
| Nes | TGCCCTAGAGACGGTGTCTCA | AATCGCTTGACCTTCCTCCC |  | 94 |
| Glul | CATCCGTCCCCCTCTGTGT | GAGGACCAAGAAATGCCAACTT |  | 94 |
| Sox2 | AAAAAACCACCAATCCCATCC | CCCCCAAAAAGAAGTCCCAA |  | 90 |
| Fgfr3 | GGCCCTTTTTGACCGAGTCTA | CCCAGCGTAAAGATCTCCCAG |  | 81 |
| Aldh1l1 | ATTCCCAAGGGTGTGGTCAAC | CATCAGGGTGGTCTGAGAGTCTCT |  | 76 |
| Glt1/Slc1a2 | GCGCATGTGCGACAAGCTGG | GCGATGCCAAGCGAAGCAGC |  | 99 |
| Glast1/Slc1a3 | GAAGCCATCATGCGATTGG | CAACAATCTTCCCTGCGATCA |  | 82 |
| S100β | GGGTGACAAGCACAAGCTGA | TCCACCACTTCCTGCTCCTT |  | 99 |
| Rn18s | GGACCAGAGCGAAAGCATTT | TCGTCTTCGAACCTCCGACTT |  | 71 |
| Actb | GCTCCTCCTGAGCGCAAG | CATCTGCTGGAAGGTGGACA |  | 75 |
| Gapdh | TGCACCACCAACTGCTTAGC | GGCATGGACTGTGGTCATGA |  | 87 |
| Hprt | ATGGGAGGCCATCACATTGT | ATGTAATCCAGCAGGTCAGCAA |  | 77 |
| Ef1a2 | CTGGATGCTCGCCATCAAA | GGCGCTTTTCCTCTTGAAGAA |  | 52 |
| * lacks sensitivity | | | | |
